# Heart-Lung Interactions During Mechanical Ventilation: Analysis via a Cardiopulmonary Simulation Model

**DOI:** 10.1109/OJEMB.2021.3128629

**Published:** 2021-11-17

**Authors:** Nikolaos Karamolegkos, Antonio Albanese, Nicolas W. Chbat

**Affiliations:** ^1^ Columbia University 5798 New York NY 10027 USA; ^2^ Columbia University 5798 New York NY 10027 USA; Quadrus Medical Technologies White Plains NY 10607 USA

**Keywords:** Cardiopulmonary Model, Heart-Lung Interactions, Mechanical Ventilation

## Abstract

Heart-lung interaction mechanisms are generally not well understood. Mechanical ventilation, for example, accentuates such interactions and could compromise cardiac activity. Thereby, assessment of ventilation-induced changes in cardiac function is considered an unmet clinical need. We believe that mathematical models of the human cardiopulmonary system can provide invaluable insights into such cardiorespiratory interactions. In this article, we aim to use a mathematical model to explain heart-lung interaction phenomena and provide physiologic hypotheses to certain contradictory experimental observations during mechanical ventilation. To accomplish this task, we highlight three model components that play a crucial role in heart-lung interactions: 1) pericardial membrane, 2) interventricular septum, and 3) pulmonary circulation that enables pulmonary capillary compression due to lung inflation. Evaluation of the model’s response under simulated ventilation scenarios shows good agreement with experimental data from the literature. A sensitivity analysis is also presented to evaluate the relative impact of the model’s highlighted components on the cyclic ventilation-induced changes in cardiac function.

## I. Introduction

The human body is a complex dynamic system with sophisticated neurohumoral control mechanisms. Besides autonomic and humoral regulatory processes, direct mechanical heart-lung interactions also exist. These arise from to the fact that the heart resides within the thoracic cavity. Respiratory activity causes cyclic variations in lung volume and in intrathoracic (pleural) pressure. Such variations are, in turn, transferred to all cardiovascular structures within the thoracic cavity, such as thoracic veins, heart, pulmonary circulation, and aorta, thus leading to cyclic changes in cardiac function. These respiratory-induced cardiac variations appear in normal breathing (pulsus paradoxus) [1], but they become accentuated in mechanically ventilated subjects under positive pressure ventilation (reversed pulsus paradoxus) [2].

Positive pressure ventilation (PPV) is a life support therapy that is typically instituted when a patient is unable to maintain adequate ventilation on their own. It is estimated that, even before the pandemic, every year nearly 1.5 million patients across the United States require some form of mechanical ventilation support [3], [4] and this number is set to increase. Despite the undoubted benefits of this therapy, PPV may cause adverse consequences as a result of the aforementioned mechanical effects of respiration on cardiac function. Selection of inappropriate ventilator settings, such as elevated pressure support or positive end-expiratory pressure (PEEP) levels, can induce substantial changes in pleural pressure and potentially compromise cardiac performance.

Balancing such ventilatory interventions requires a comprehensive understanding of the interactions between the different components of the cardiopulmonary system. These interactions can be captured 1) by black-box models, where input-output relationships are described by some form of mathematical representations without a direct physiological interpretation [5], or 2) by physiology-based mathematical models which incorporate a mechanistic description of the system being modeled. In this paper, we focus on this latter category of modeling approaches as we believe that this is better suited to the understanding of cardio-respiratory interactions. Model-based computer simulations could be effective tools to conduct 1) virtual physiological experiments, 2) analyze cardiopulmonary dynamics, 3) investigate different clinical scenarios, and 4) assess the outcomes of specific treatments [6].

Over the past few years, several investigators [7]–[10] have proposed mathematical models of the integrated cardiorespiratory physiology. However, most of the earlier work was not tailored to simulate mechanical ventilation scenarios. The model proposed by Cheng *et al.* [9], though comprehensive, is primarily focused on the response of the autonomic nervous system (ANS) during sleep, like Cheyne-Stokes respiration and sleep apnea. The model from Lu *et al.* [10], on the other hand, has more rigor in describing the dynamics associated with mechanical heart-lung interactions. However, this model is less detailed in the description of the short-term neural mechanisms that are involved in the cardiovascular and respiratory control systems. The integrated cardiopulmonary model (CP Model) that was recently introduced by our group [7], [8] features all major cardiorespiratory control mechanisms as well as cardiovascular circulation, respiratory mechanics, alveolar and tissue gas exchange, and gas transport. However, despite its rigor in neural pathways, the CP Model presented limitations in mechanical heart-lung interaction mechanisms, as highlighted by Albanese *et al.* [7].

Experimental studies [11] have shown that the cyclic respiratory-induced changes in cardiac activity are predominantly attributed to four elements: thoracic cavity, pericardial membrane (pericardium), interventricular septum, and pulmonary peripheral vessels whose resistance to blood flow changes as a function of alveolar volume. In this paper, we 1) incorporate an enhanced cardiac model with septum and pericardium into the original CP Model, and 2) revise the pulmonary circulation model to include a varying pulmonary peripheral resistance as a function of alveolar volume.

The manuscript is organized as follows. First, in the Methods section, we describe the CP Model and highlight the pericardial membrane, interventricular septum, and pulmonary circulation model. Second, in the Results & Validation section, we demonstrate the model’s validity by comparing its response during simulated ventilation conditions against experimental data from mechanically ventilated subjects. We also perform a sensitivity analysis to evaluate the relative impact of the model’s highlighted components to variations in cardiac function that are induced by mechanical ventilation. Additionally, we provide physiologic explanations to contradictory experimental results. Lastly, in the Discussion section, we discuss the limitations of such a model and outline future improvements.

## II. Methods

### A. The Cardiopulmonary Model

**Figure 1.**
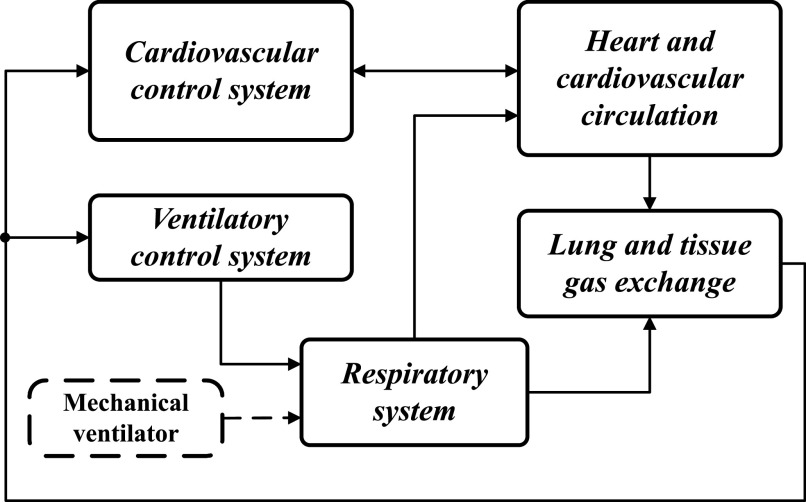
High-level block diagram of the cardiopulmonary model.

Fig. 1 shows the high-level block diagram of the cardiopulmonary model (CP Model). The original CP Model was developed by Albanese *et al.* [7] using data from healthy, spontaneously breathing, individuals and validated under hypoxia and hypercapnia by Cheng *et al.* [8], while Karamolegkos *et al.* [12] improved the model by including the Hering-Breuer reflex. Model parameters were assigned in reference to a generic 70-kg healthy subject.

### B. Modeling Highlights

**Figure 2.**
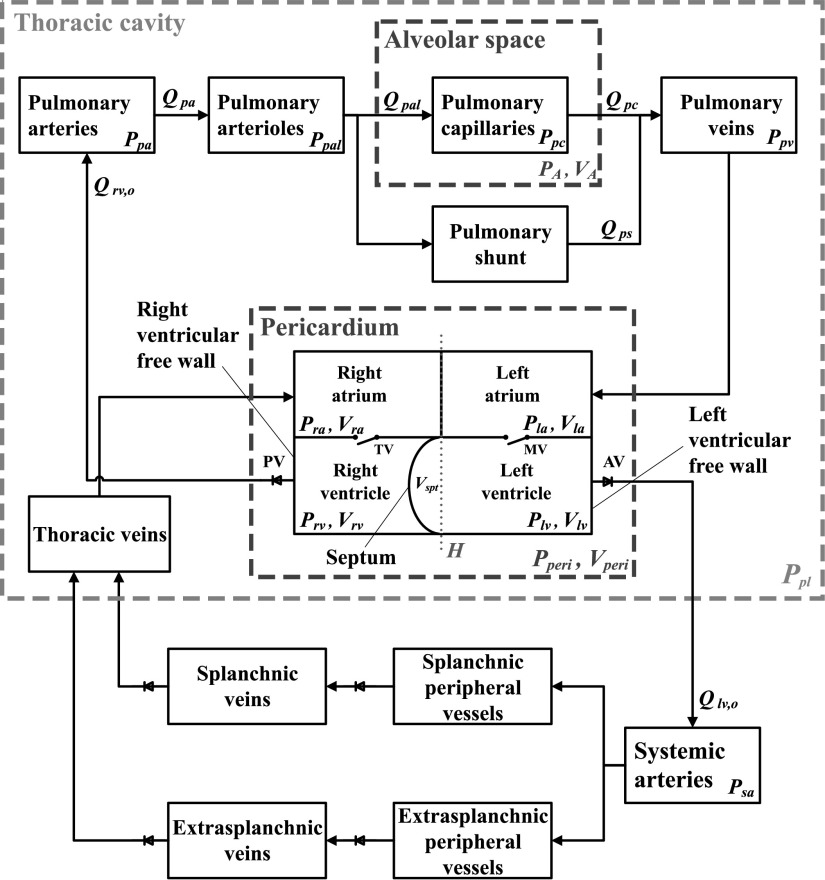
Schematic block diagram of the cardiovascular system of the enhanced CP Model. $P_{sa}$ and $P_{pa}$, systemic and pulmonary arterial blood pressures; $P_{ra}$ and $P_{la}$, right and left atrial pressures; $P_{rv}$ and $P_{lv}$, right and left ventricular pressures; $P_{pal}$, pulmonary arteriolar pressure; $P_{pc}$, pulmonary capillary pressure; $P_{pv}$, pulmonary venous pressure; $P_{peri}$, pericardial pressure; $P_{pl}$, pleural (intrathoracic) pressure; $P_{A}$, alveolar pressure; $V_{ra}$ and $V_{la}$, right and left atrial volumes; $V_{rv}$ and $V_{lv}$, right and left ventricular volumes; $V_{spt}$, septal volume; $V_{peri}$, pericardial volume; $V_{A}$, alveolar volume; $Q_{rv,o}$ and $Q_{lv,o}$, right and left ventricular output blood flows; $Q_{pa}$, pulmonary arterial blood flow; $Q_{pal}$, pulmonary arteriolar blood flow; $Q_{pc}$, pulmonary capillary blood flow; $Q_{ps}$, pulmonary shunt blood flow; MV, mitral valve; AV, aortic valve; TV, tricuspid valve; PV, pulmonary valve; $H$, imaginary plane defining the volumes of the septum and of the right and left ventricular free walls.

Fig. 2 presents the cardiovascular system components that are important for studying heart-lung interactions during mechanical ventilation. Detailed description of the original CP Model can be found in [7], while we now present the details of the cardiovascular system components which gave rise to an enhanced CP Model (Fig. 2) and allowed us to study heart-lung interactions.

#### 1) Pericardium

The pericardium is the membrane that contains the heart and serves as the connective medium between the heart and the chest wall. The mechanical behavior of the pericardium resembles that of a passive fluid chamber with nonlinear elastic properties. Thus, its pressure-volume characteristics are modeled with an exponential function as proposed by Chung *et al.* [13]. Such a function relates the transmural pressure across the pericardium ($P_{pcd}$) to the total blood volume ($V_{tot}$) enclosed by it as

\begin{equation*}
P_{pcd}(V_{tot}) = P_{0, pcd} \cdot \left(e^{k_{E,pcd} \cdot (V_{tot} - V_{u, pcd})} - 1 \right), \tag{1}
\end{equation*}

where $P_{pcd}$ is the difference between the pressure inside the pericardial membrane ($P_{peri}$) and the pleural pressure ($P_{pl}$) outside of it, $P_{0,pcd}$ is a scaling factor, $k_{E,pcd}$ is an elastance coefficient, and $V_{u, pcd}$ is the volume enclosed by the pericardium when the transmural pressure $P_{pcd}$ is zero (unstressed volume). The values of the parameters in (1) have been adopted from [10] and are reported in Table I. $V_{tot}$ comprises the volumes of all four heart chambers and the volume of fluid within the pericardial space ($V_{peri} = 40\ \text{ml}$ [14]). The volumes of the myocardial tissue and coronary circulation are neglected in this model.

**TABLE I:** Parameters of the Heart Model in Basal Conditions

Parameter	RV free wall	LV free wall	Pericardium	Septum
$k_{E, (\cdot)}$ (ml$^{-1}$)	0.011 [15]	0.014 [15]	0.005 [10]	0.175 [16]
$P_{0, (\cdot)}$ (mmHg)	1.5 [15]	1.5 [15]	0.5 [10]	1.11 [16]
$V_{u, (\cdot)}$ (ml)	35.904 [7]	14.758 [7]	200 [10]	0 [16]
$E_{max, (\cdot)0}$ (mmHg/ml)	1.412 [17]	2.392 [17]	—	32.4

Note that the subscript $(\cdot)$ indicates the respective compartment, namely $rvf$ for the right (RV) ventricular free wall, $lvf$ for the left ventricular (LV) free wall, $pcd$ for the pericardium, and $spt$ for the septum. $k_{E}$, elastance coefficient; $P_{0}$, scaling factor; $V_{u}$, unstressed volume; $E_{max, 0}$, basal value of wall elastance at the maximum contraction point (end-systole) which is subject to changes by the autonomic nervous system.

#### 2) Interventricular Septum

The CP Model features four heart chambers. The two ventricles interact with each other due to the presence of the interventricular septum, whereas the two atria are assumed to be connected through a rigid wall since atrial interference has a minimal contribution to the overall cardiovascular hemodynamics.

To describe the interventricular septum, we follow the model proposed by Chung *et al.* [13] which was validated with echocardiographic images. An imaginary plane $H$ is assumed to split the total ventricular space into three functional volumes (see Fig. 2): a right ventricular free wall volume ($V_{rvf}$), a left ventricular free wall volume ($V_{lvf}$), and a septal volume ($V_{spt}$). Each one of these three volumes represents blood volume that is bounded by the corresponding wall (namely, left and right ventricular free walls and septal wall, respectively) and the plane $H$. Furthermore, due to the natural position of the interventricular wall protruding into the right ventricle (Fig. 2), right and left ventricular volumes are defined as $V_{rv} = V_{rvf} - V_{spt}$ and $V_{lv} = V_{lvf} + V_{spt}$, respectively.

Heart contraction is simulated by the activation of the three walls delineated above. Their contractile activities are modeled by means of variable-elastance models such that the pressure-volume relationships vary between end-systolic and end-diastolic states. The transition between end-systolic and end-diastolic states is governed by a half-sine activation function $\phi (t)$ whose period is equal to the heart period [7]. The pressure-volume relationships of the two ventricular free walls remain as in [7]. For instance, the maximal isometric transmural pressure across the left ventricular free wall ($P_{max, lvf}$) is defined as a function of $V_{lvf}$ according to the equation

\begin{align*}
P_{max, lvf}(t) =& \phi (t) \cdot P_{max, lvf}(V_{lvf})|_{ES} \\
& + \left(1 - \phi (t)\right) \cdot P_{max, lvf}\left(V_{lvf}\right)|_{ED}, \tag{2}
\end{align*}

where $P_{max, lvf}(V_{lvf})|_{ES} = E_{max, lvf} \cdot (V_{lvf} - V_{u, lvf})$ and $P_{max, lvf}(V_{lvf})|_{ED} = P_{0, lvf} \cdot (e^{k_{E, lvf} \cdot V_{lvf}} - 1)$ are the end-systolic ($ES$) and end-diastolic ($ED$) pressure-volume relationships, respectively; these determine the elastic behavior of the free wall during a cardiac cycle. $E_{max, lvf}$ is the wall elastance at the maximum contraction point (end-systole), $V_{u, lvf}$ is the unstressed volume of the ventricular wall, and $P_{0, lvf}$, $k_{E, lvf}$ are the parameters that characterize the end-diastolic exponential function. The maximal pressure of the left ventricle (prior to any viscous losses due to blood flow over the aortic valve) can be computed by taking into account $P_{peri}$, which acts as the external (reference) pressure of the ventricular free wall. Hence, $P_{max, lv} = P_{max, lvf} + P_{peri}$. For the sake of brevity, the equations to simulate ventricular function, including ventricular filling, valve operation, and ventricular ejection, are omitted from this article. A detailed description of these elements can be found in [15].

As for the elastic properties of the right ventricular free wall and septum, an analogous approach is considered. The right ventricular free wall adheres to the same formulation of the biphasic pressure-volume relationship as in (2). On the other hand, the behavior of the septal wall is modeled via a nonlinear volume-pressure relationship similar to the approach followed in [13]. The values of the parameters that characterize the elastic properties of all three cardiac walls are reported in Table I, along with the corresponding reference sources.

Note that $E_{max, (\cdot)0}$ in Table I indicates the basal elastance value that is modulated by the autonomic nervous system (ANS), whereas $E_{max, (\cdot)}$, like the one used in (2), is the resultant elastance value due to the ANS action. This is so, because the ANS efferent sympathetic pathway regulates the magnitude of cardiac contraction by controlling the elastance values of the two ventricular free walls and of the septum. The equations describing the ANS actions on the ventricular free wall elastances are kept the same as the ones in [7], which were taken from Ursino and Magosso [17]. As for the septal elastance, we follow the same approach as in [17]. The equations are reported in the Appendix.

#### 3) Pulmonary Circulation

**Figure 3.**
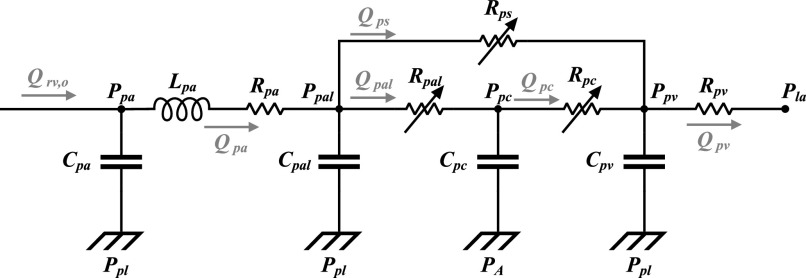
Electrical diagram of the pulmonary circulation model of the CP Model. $P$, air/blood pressure; $Q$, blood flow; $R$, resistance; $L$, inertance; $C$, capacitance (compliance). Subscripts: $pa$, pulmonary arteries; $pal$, pulmonary arterioles; $pc$, pulmonary capillaries; $pv$, pulmonary veins; $la$, left atrium; $ps$, pulmonary shunt; $pl$, pleural space; $A$, alveolar space. The variable resistances $R_{pal}$, $R_{pc}$, and $R_{ps}$ are indicated by diagonal arrows.

Fig. 3 shows the pulmonary circulation model that is developed based on the work by Lu *et al.* [10]. The model consists of four pressure nodes (pulmonary arteries ($P_{pa}$), pulmonary arterioles ($P_{pal}$), pulmonary capillaries ($P_{pc}$), and pulmonary veins ($P_{pv}$)), a pulmonary shunt compartment, and three variable resistances (pulmonary arteriolar (or pre-capillary) resistance ($R_{pal}$), pulmonary shunt resistance ($R_{ps}$), and pulmonary post-capillary resistance ($R_{pc}$)). By considering two nodes ($P_{pal}$ and $P_{pc}$) for the pulmonary peripheral compartment, we achieve an explicit separation between the extra-alveolar peripheral vessels (arterioles) and the capillary vessels that are near the alveoli. Pleural pressure ($P_{pl}$) can then be set as the external pressure of the pulmonary arterioles (node $P_{pal}$ in Fig. 3), while the pressure at the pulmonary capillaries (node $P_{pc}$ in Fig. 3) is referenced to alveolar pressure ($P_{A}$). This configuration follows experimental evidence that the extravascular pressure of the capillaries that participate in gas exchange resembles alveolar rather than pleural pressure [11]. Additionally, this configuration allows a more accurate representation of the pulmonary shunt. Anatomically, the shunt is located between the pulmonary arteries and the pulmonary veins and comprises the pulmonary peripheral vessels that do not participate in gas exchange. Hence, it is more reasonable to model the shunt as a compartment that originates from the arteriolar pressure node and is parallel to the pulmonary capillaries (see Fig. 3).

Next, we seek to capture the physiology of the compression of the pulmonary capillaries due to lung expansion [18]. Such an interaction is considered the primary factor for the increase in pulmonary impedance (thus, in right ventricular afterload), typically observed during inspiration in positive pressure ventilation [19]. To capture this phenomenon, we introduce a variable resistance ($R_{pal}$) that changes as a function of alveolar volume ($V_A$) and shunt fraction ($sh$):

\begin{equation*}
R_{pal}(V_A, sh) = \frac{R_{pp, tot}}{2 \cdot (1 - sh)} \cdot \left(\frac{V_A}{FRC_{nom}} \right)^2, \tag{3}
\end{equation*}

where $R_{pp, tot}$ is the total resistance of the pulmonary peripheral circulation at steady-state conditions and at a nominal functional residual capacity ($FRC_{nom}$),

\begin{equation*}
R_{pp, tot} = \frac{R_{ps} \cdot \left(R_{pc} + R_{pal@FRC_{nom}} \right)}{R_{ps} + R_{pc} + R_{pal@FRC_{nom}}}. \tag{4}
\end{equation*}

The nominal functional residual capacity ($FRC_{nom}$) is here calculated to be 2.25 liters for a nominal set of respiratory system parameters (airway resistance of 1.7459 cmH$_{2}$O$\cdot$s/l, and lung and chest wall compliances of 0.2 l/cmH$_{2}$O and 0.2445 l/cmH$_{2}$O, respectively). The complete set of equations that describe the pulmonary circulation model is presented in the Appendix.

Table II presents the parameters of the pulmonary circulation model of Fig. 3 along with their reference sources. Note that the compliance and unstressed volume values of the pulmonary arterioles and pulmonary capillaries ($C_{pal}$ and $V_{u, pal}$, and $C_{pc}$ and $V_{u, pc}$, respectively) have been computed such that their parallel arrangement (assuming zero-flow conditions) provides values equivalent to those published in [7] and [17]; namely, $C_{pal} + C_{pc} = 5.8$ ml/mmHg, which is indeed the compliance of the pulmonary peripheral compartment in [17], and $V_{u, pal} + V_{u, pc} = 108.24$ ml, which is the unstressed volume of the pulmonary peripheral circulation in [7]. To complete the calculation, we follow the convention used by Lu *et al.* [10] where $C_{pc} = 2 \cdot C_{pal}$ and $V_{u, pc} = \frac{3}{2} \cdot V_{u, pal}$.

**TABLE II:** Parameters of the Pulmonary Circulatory System

Compliance (ml/mmHg)	Unstressed volume (ml)	Resistance (mmHg$\cdot$s/ml)	Inertance (mmHg$\cdot$s$^{2}$/ml)
$C_{pa} = 0.76$ [7]	$V_{u, pa} = 0$ [7]	$R_{pa} = 0.023$ [7]	$L_{pa} = 1.8 \cdot 10^{-4}$ [7]
$C_{pal} = 1.93$	$V_{u, pal} = 43.30$	$R_{pp, tot} = 0.0894$ [17]	
$C_{pc} = 3.87$	$V_{u, pc} = 64.94$		
$C_{pv} = 25.37$ [7]	$V_{u, pv} = 105.6$ [7]	$R_{pv} = 0.0056$ [7]	

See text and Fig. 3 legend for explanation of symbols.

## III. Results & Validation

In this section, we demonstrate the capability of the CP Model in 3 steps. As a first step, we prove the model’s validity during normal resting conditions. As a second step, we perform three validation studies with real patient data followed by a sensitivity analysis, and as a third step we use the model to explain important physiological phenomena. A stability analysis of the dynamic model was omitted from this paper for brevity.

The dynamic equations of the CP Model were programmed in Simulink (MathWorks, Natick, MA). Simulation results presented in the following sections were obtained using the fourth-order Runge-Kutta method with a fixed-size integration step of 0.0005 seconds (2 kHz rate).

### A. Results in Normal Resting Conditions

Table III presents the static values (at end-expiration) of the main hemodynamic variables predicted by the CP Model in normal resting conditions, along with the normal ranges observed in the general population. All variables fell within expected normal physiological ranges.

**TABLE III:** Static Values of Main Hemodynamic Variables in Normoxic Conditions

Variable	Model	Normal range
*Systemic arterial pressure*, $P_{sa}$ (mmHg)		
Mean	89.39	70–105 [7]
Systolic	121.85	100–140 [7]
Diastolic	76.77	60–90 [7]
*Left ventricular pressure*, $P_{lv}$ (mmHg)		
Systolic	121.85	90–140 [7]
End-diastolic	4.65	4–12 [7]
*Left ventricular volume*, $V_{lv}$ (ml)		
End-systolic	55.98	37–57 [20]
End-diastolic	136.07	121–163 [20]
*Left atrial pressure*, $P_{la}$ (mmHg)		
Mean	4.35	4–12 [21]
*Pulmonary arterial pressure*, $P_{pa}$ (mmHg)		
Mean	14.35	9–18 [21]
Systolic	26.86	15–28 [7]
Diastolic	7.11	5–16 [7]
*Right ventricular pressure*, $P_{rv}$ (mmHg)		
Systolic	26.86	15–28 [7]
End-diastolic	1.91	0–8 [7]
*Right ventricular volume*, $V_{rv}$ (ml)		
End-systolic	49.31	36–64 [22]
End-diastolic	127.05	121–167 [22]
*Right atrial pressure*, $P_{ra}$ (mmHg)		
Mean	1.71	2–6 [7]

The model-predicted values are taken from the end-expiratory heart beat after a 2,000-second simulation.

### B. Validation in Mechanical Ventilation Conditions

Validation of complex physiological models is a challenging task due to the high number parameters and variables and there has not been an established quantitative approach for validating such models [6], [23]. Nevertheless, Summers *et al.* [23] have proposed the following set of qualitative criteria that we have adopted in this work: the model predictions are in good agreement with experimental data when the simulated outputs 1) are directionally appropriate in a qualitative manner, 2) have steady-state values that closely match the experimental data, and 3) are fairly accurate during the transient dynamic state of the system’s response.

In this section, we aim to validate the CP Model in mechanical ventilation conditions according to the above criteria. To do so, we leverage data from three different human studies that evaluate the hemodynamic effects of mechanical ventilation during 1) changes in positive pressure ventilation (PPV) in spontaneously breathing healthy subjects [24], 2) step changes in PEEP in sedated patients [25], and 3) constant ventilatory support in sedated patients for investigating the cyclic ventilation-induced changes in cardiac function [26]. As our main goal is to show that the model is able to describe the physiology of an average patient population, rather than matching a specific patient dataset, no quantitative metric of the goodness of fit between simulated and experimental data, such as a root mean squared error, is considered.

For the simulations presented hereafter, a few parameters were adjusted from their nominal values reported in Table III. Such parameter adjustments were necessary because the baseline cardiorespiratory variables of the subjects in the three aforementioned human studies differed from their average normal values. Possible factors include the age distribution of the study population or the disease state characterizing those subjects. Note, however, that parameters were modified at the beginning of each simulation and subsequently kept constant for the duration of the simulation. This further justifies the authors’ choice not to use any goodness of fit metric for evaluating the model’s performance against experimental data.

#### 1) Validation study 1: Changes in PPV in spontaneously breathing healthy subjects

**Figure 4.**
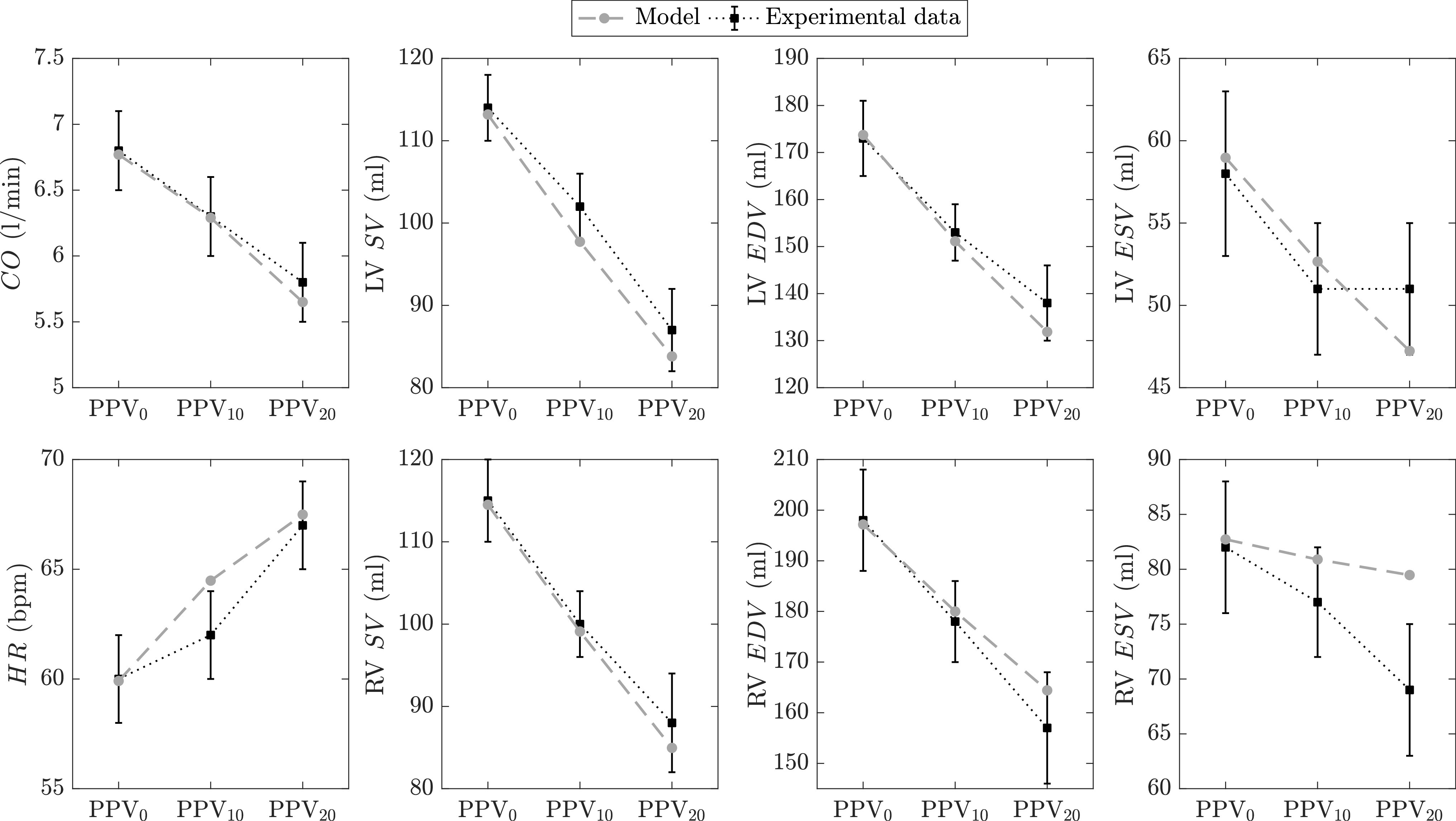
Cardiovascular response to step changes in the level of positive pressure ventilation (PPV). Experimental data (*black* squares and error bars are means and standard errors of the means, respectively) collected during a human study with 18 spontaneously breathing healthy subjects as published by Kyhl *et al.* [24]. Both model-simulated (*gray* circles) and experimental data are assessed at end-expiratory heart beats at the end of each 30-minute PPV period. LV, left ventricular; RV, right ventricular; $CO$, cardiac output; $HR$, heart rate (bpm, beats per minute); $SV$, stroke volume; $EDV$, end-diastolic volume; $ESV$, end-systolic volume.

Our first validation study [24] includes data from 18 healthy volunteers who underwent 3 consecutive 30-minute phases under different ventilation regimes while actively breathing: *phase 1*, normal conditions with no positive pressure ventilation (labeled as PPV$_{0}$); *phase 2*, ventilation with pressure support of 3 cmH$_{2}$O and PEEP of 10 cmH$_{2}$O (labeled as PPV$_{10}$); *phase 3*, ventilation with pressure support of 3 cmH$_{2}$O and PEEP of 20 cmH$_{2}$O (labeled as PPV$_{20}$). Magnetic resonance imaging was used to measure the volumes of the heart chambers of the study volunteers during each phase. Fig. 4 shows the comparison between experimental data and model predictions in terms of cardiac output ($CO$), heart rate ($HR$), left (LV) and right (RV) ventricular stroke volumes ($SV$), end-diastolic volumes ($EDV$), and end-systolic volumes ($ESV$).

The baseline values (at PPV$_{0}$) of $SV$ and $EDV$ of the study volunteers were abnormally high compared to the average population values in Table III. Hence, the following parameter adjustments were applied: $E_{max, lvf0}$ was set to 3.05 mmHg/ml, $E_{max, rvf0}$ was set to 0.8 mmHg/ml, $k_{E, lvf}$ was set to 0.008 ml$^{-1}$, and $k_{E, rvf}$ was set to 0.007 ml$^{-1}$.A possible explanation for the high experimental $SV$ and $EDV$ could be a low average age of the study population (16–71 years). In fact, based on the studies by Maceira *et al.* [20], [22], end-diastolic and stroke volumes are inversely related to age, with younger subjects exhibiting higher cardiac volumes due to higher ventricular compliances (i.e., low elastance coefficients $k_{E}$).

The simulation results in Fig. 4 show that our model is able to mimic the effects of step changes in PPV on cardiac activity. All experimental and simulated cardiac volumes are reduced during positive pressure ventilation (see PPV$_{10}$ and PPV$_{20}$), while heart rate increases due to sympathetic activation. Despite the increase in $HR$, left ventricular $SV$ markedly drops as PPV is increased, thus leading to a reduction in cardiac output. The drop in $CO$ is expected since systemic venous return is reduced due to the increase in pleural pressure following the step changes in PPV. In addition, model predictions, except RV $ESV$, are within one standard deviation of the sampling distribution (standard error) of the subjects for all PPV levels. Although simulated RV $ESV$ changes are directionally in agreement with those reported in [24], their magnitude is small, especially at PPV$_{20}$. Such a discrepancy between model and experimental RV $ESV$ at PPV$_{20}$ could be due to a disproportionately high model-predicted right ventricular afterload when PPV is increased which causes a high RV $ESV$ value. Right ventricular afterload is indeed directly associated with pulmonary capillary collapse, which is shown to be affected by the respiratory system’s mechanical properties (more detailed explanation is provided in the *Explanation of physiological phenomena* section).

#### 2) Validation Study 2: Step Changes in PEEP on Sedated Patients

**Figure 5.**
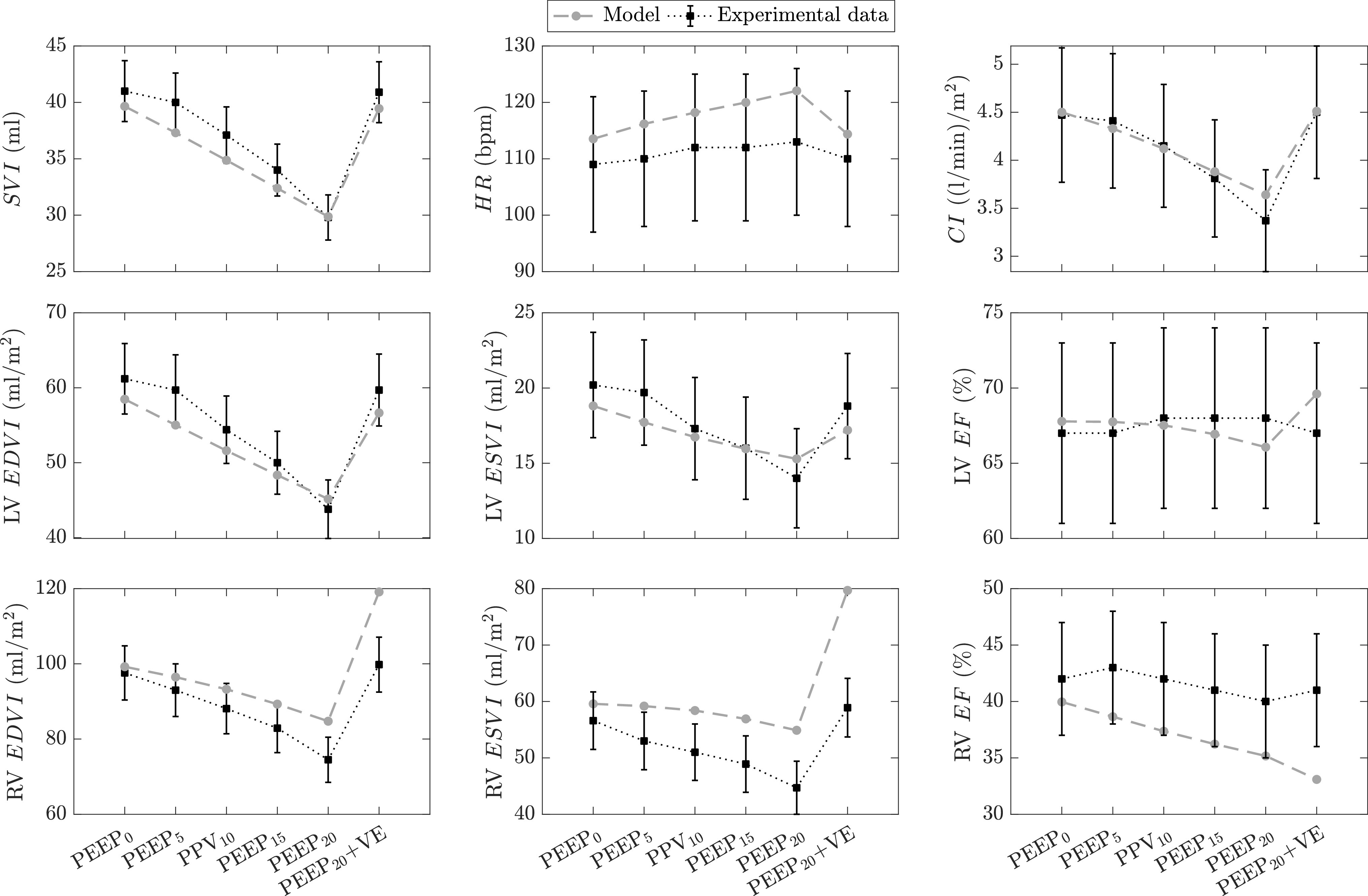
Cardiovascular response to step changes in positive end-expiratory pressure (PEEP) and blood volume expansion (VE) in ARDS subjects. Experimental data (*black* squares, and error bars are means and standard errors of the means, respectively) as reported by Dhainaut *et al.* [25] from a human study with 8 ARDS patients. Each PEEP level is applied for 20 minutes. Both model-simulated (*gray* circles) and experimental data are computed at end-expiratory heart beats during the last 10 minutes of each PEEP segment. The model’s volumetric indices are normalized by assuming a nominal body surface area of 1.9 m$^{2}$. PEEP$_{x}$, PEEP at x cmH$_{2}$O where x = {0, 5, 10, 15, 20}; PEEP$_{20}$ + VE, PEEP at 20 cmH$_{2}$O and blood volume expansion with 625$\pm$72 ml of plasma expanders; $SVI$, stroke volume index; $HR$, heart rate (bpm, beats per minute); $CI$, cardiac index; $EDVI$, end-diastolic volume index; $ESVI$, end-systolic volume index; $EF$, ejection fraction; LV, left ventricular; RV, right ventricular.

Our second validation study [25] analyzes the cardiovascular response to step changes in PEEP and blood infusion (volume expansion, VE) in 8 ARDS patients. The results are summarized in Fig. 5, where model simulation results are compared against the corresponding experimental data in terms of cardiac index ($CI$), $HR$, stroke volume index ($SVI$), LV and RV end-diastolic ($EDVI$) and end-systolic ($ESVI$) volume indices, and ejection fractions ($EF$). All volumetric indices are normalized by the body surface area ($BSA$). According to the study in [25], PEEP was adjusted from 0 to 20 cmH$_{2}$O in increments of 5 cmH$_{2}$O (PEEP$_{0}$, PEEP$_{5}$, PEEP$_{10}$, PEEP$_{15}$, and PEEP$_{20}$). At PEEP$_{20}$, plasma expanders (VE) were administered in order to normalize the cardiac index at a level similar to baseline (PEEP$_{0}$). Each ventilatory period lasted 20 minutes and the experimental data in Fig. 5 were reported as the average values over the last 10 minutes. All other ventilator settings, besides PEEP, were kept unchanged throughout the study, with tidal volume set to 10–14 ml/kg of body weight and $F{\scriptstyle I}_{O_2}$ set to 50%. No medications were administered to the subjects during the study.

In our simulation, the experimental basal values of $EDVI$ and $ESVI$ were matched by initially setting $E_{max, lvf0}$, $E_{max, rvf0}$, $k_{E, lvf}$, and $k_{E, rvf}$ to 4 mmHg/ml, 0.4 mmHg/ml, 0.007 ml$^{-1}$, and 0.0065 ml$^{-1}$, respectively. Such parameter changes were justified because of the study population’s low average age (36 years). In order to match the elevated heart rate reported in [25], the basal heart period ($T_0$) of the model was adjusted, from its nominal value of 0.58 seconds [7], [15] to 0.27 seconds. Additionally, the basal values of systemic and pulmonary peripheral resistances (splanchnic peripheral resistance, $R_{sp,0}$ = 1 mmHg$\cdot$s/ml, extrasplanchnic resistance, $R_{ep,0}$ = 0.5 mmHg$\cdot$s/ml, and total pulmonary peripheral resistance, $R_{pp,tot}$ = 0.23 mmHg$\cdot$s/ml) were changed in order to match the data in [25], i.e., a systemic and a pulmonary vascular resistance of 0.51 mmHg$\cdot$s/ml and 0.16 mmHg$\cdot$s/ml, respectively. As for the respiratory system, we adjusted the lung and chest wall mechanical properties to be: $C_L = 0.065$ l/cmH$_{2}$O, $C_{cw} = 0.1$ l/cmH$_{2}$O, $V_{u,L} = 0.4$ l, and $V_{u, cw} = 1$ l. These initial changes allowed us to achieve a tidal volume of about 0.75 liters (about 11 ml/kg of body weight) and a basal mean $P_{pl}$ value of -1.07 cmH$_{2}$O, compared to 0$\pm$1.6 cmH$_{2}$O in [25]. Also, since the experimental data were obtained from sedated patients, the ventilation control model was modified by setting the sensitivities of the central and peripheral chemoreflex mechanisms as well as the basal breathing amplitude to zero. Finally, venous admixture (pulmonary shunt fraction) and $F{\scriptstyle I}_{O_2}$ were set to 0.35 (35%) and 50%, respectively, as reported by Dhainaut *et al.* [25].

The results in Fig. 5 show that the model captures well the effect of PEEP on the overall cardiac function. Left and right ventricular $EDVI$ are reduced as PEEP is progressively increased due to a reduction in systemic venous return. Reduction in $EDVI$ then leads to a decrease in $SVI$ as explained by the Frank-Starling mechanism. Moreover, left ventricular $EF$ is relatively constant across the entire PEEP range, indicating that LV afterload is not significantly affected by PEEP application. In contrast, right ventricular $EF$ is lower at high PEEP values in both experimental and simulation results. This effectively demonstrates an elevated pulmonary impedance owing to the compression of the pulmonary capillaries by the PEEP-induced lung expansion. However, the response of the model to the VE protocol (PEEP$_{20}$ + VE) is somewhat in disagreement with the experimental results in [25]. While simulated left ventricular volume indexes return to basal values (PEEP$_{0}$ levels) after VE, in agreement with the experimental observations in [25], the model predicts an increase in RV $EDVI$ and $ESVI$ beyond their basal values, which is not found experimentally. Such a disparate behavior in RV $EDVI$ and $ESVI$ after VE has also been exhibited in human studies. The study under investigation [25] shows a complete return of RV $EDVI$ to baseline. However, an earlier study from Dhainaut *et al.* [27] demonstrated a marked increase in right ventricular afterload and hence RV $EDVI$, like what our model predicts. We therefore conjecture, just as the investigators in [27] stipulate, that model simulations indicate right ventricular overload (i.e., increase in afterload) due to the collapse of the pulmonary capillaries when PEEP is increased.

#### 3) Validation Study 3: Constant Ventilatory Support on Sedated Patients

Our third validation study [26] examines the cyclic respiratory-induced variations in left and right ventricular functions during positive pressure ventilation. The study is performed on 31 sedated patients who were mechanically ventilated under a pressure-control mode with tidal volume of 7–9 ml/kg, respiratory rate of 15 breaths/minute, end-inspiratory pause of 0.5 seconds, and PEEP of 5 cmH$_{2}$O. During the study, hemodynamic measurements were acquired via transesophageal echocardiography and the evolutions of beat-to-beat $SVI$, $EDVI$, and $ESVI$ during a breathing cycle were analyzed.

To match the baseline conditions of the subjects in the study, some parameters of the cardiovascular and respiratory systems in our model were modified. Namely, we set $C_L = 0.06$ l/cmH$_{2}$O, $C_{cw} = 0.11$ l/cmH$_{2}$O, $V_{u,L} = 0.4$ l, and $V_{u, cw} = 1$ l in order to get: a total respiratory system compliance of 0.039 l/cmH$_{2}$O (the average compliance reported in [26] is 0.038$\pm$0.007 l/cmH$_{2}$O), a tidal volume of about 0.6 liters (about 8.5 ml/kg of body weight, compared with 7–9 ml/kg in [26]) and a pleural pressure value of −1.75 cmH$_{2}$O at the end of expiration ($P_{pl}$ = −2.04$\pm$0.14 cmH$_{2}$O in [26]). In the cardiovascular compartment, we modified the following parameters: $E_{max, lvf0}$, $k_{E, lvf}$, $k_{E, rvf}$, $P_{0, lvf}$, and $P_{0, rvf}$ to 1.9 mmHg/ml, 0.016 ml$^{-1}$, 0.011 ml$^{-1}$, 0.8 mmHg, and 1.5 mmHg, respectively. As in the previous ARDS study, we altered the model’s systemic peripheral resistances ($R_{sp,0}$ = 3 mmHg$\cdot$s/ml and $R_{ep,0}$ = 1 mmHg$\cdot$s/ml) in order to match the basal systemic vascular resistance ($SVR$) that was computed from the data in [26] as the ratio between mean arterial blood pressure ($MBP$) and cardiac output, i.e., $SVR = MBP / CO = 1.06$ mmHg$\cdot$s/ml. Finally, to simulate the effects of sympathomimetic drugs, we decreased the basal heart period to 0.35 seconds to get a basal heart rate close to the experimental value (94$\pm$13 bpm).

**Figure 6.**
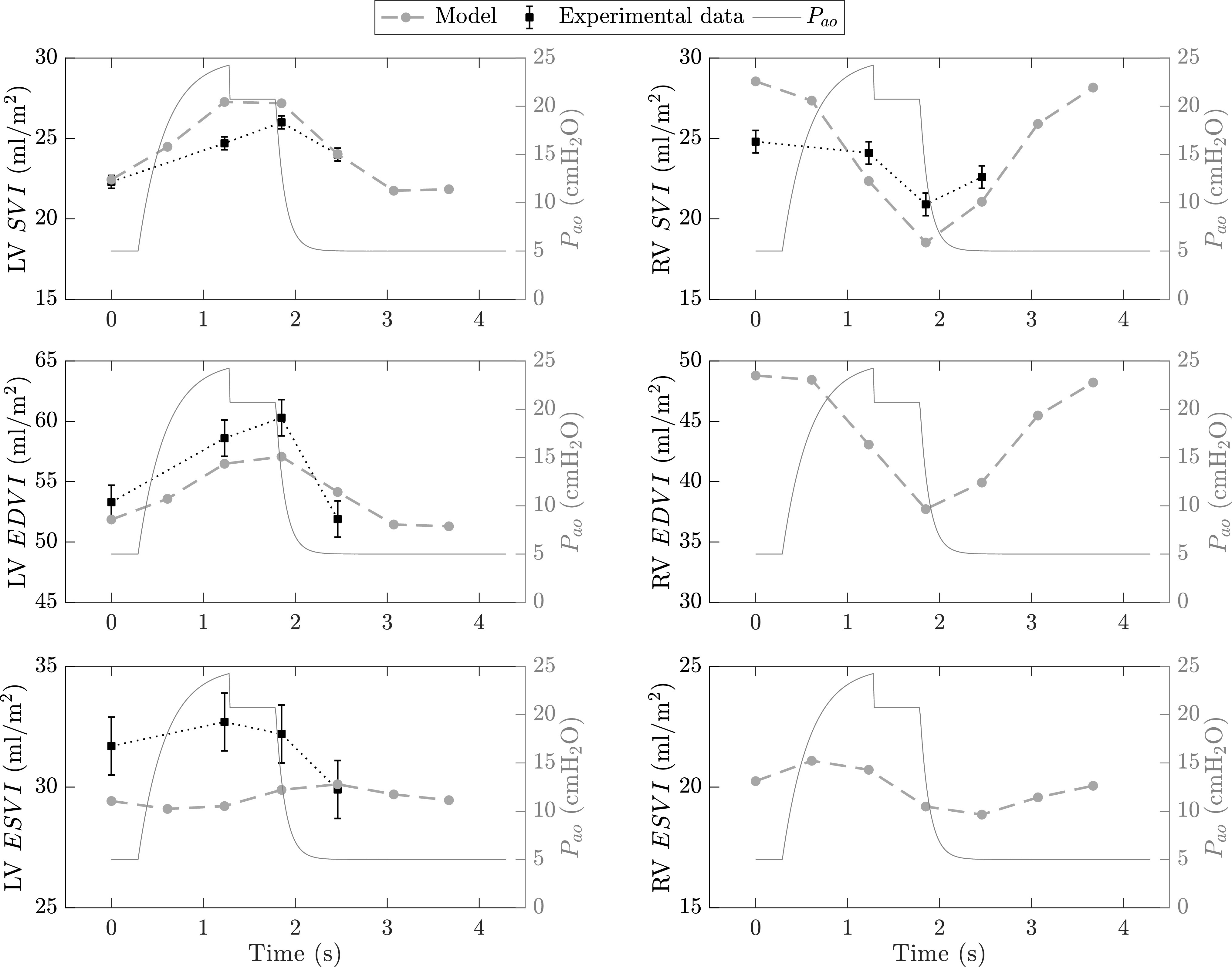
Cyclic intra-breath changes in left (*left* column) and right (*right* column) ventricular functions during positive pressure ventilation. Experimental data (*black* squares, and error bars are means and standard errors of the means, respectively) as reported by Vieillard-Baron *et al.* [26] from a study with 31 fully sedated patients under mechanical ventilation. Both model (*gray* circles) and experimental data are with reference to the left vertical axes in each subfigure. Note that [26] does not report measurements for RV $EDVI$ and $ESVI$. LV, left ventricular; RV, right ventricular; $SVI$, stroke volume index; $EDVI$, end-diastolic volume index; $ESVI$, end-systolic volume index; $P_{ao}$ (overlaid on the right vertical axes), airway opening pressure.

The results are summarized in Fig. 6 and demonstrate that the model outputs follow the trends in the experimental data for most of the indices. In particular, left ventricular $SVI$ reaches its minimum at end-expiration and its maximum at the end of inhalation. A similar trend is also observed for LV $EDVI$ as per the Frank-Starling mechanism. The increase in left ventricular $EDVI$ with inhalation is ascribed to the compression of the pulmonary peripheral vessels that promotes more blood into the left atrium and increases left ventricular filling. As to LV $ESVI$, Vieillard-Baron *et al.* [26] report a statistically insignificant change in $ESVI$ during the breathing cycle. Although the simulated variations in LV $ESVI$ are not directionally similar to the experimental data, we notice that their magnitude is considerably smaller than the intra-breath changes in $EDVI$ and $SVI$. The inspiratory decrease in LV $ESVI$ predicted by our model is nevertheless supported by other investigators [28].

The right ventricle, on the other hand, is primarily affected by changes in pleural pressure. An increase in pleural pressure during inhalation decreases systemic venous return and hence right ventricular filling (end-diastolic volume). This, in turn, leads to a reduction of right ventricular stroke volume as per the Frank-Starling mechanism. RV $SVI$ reaches its minimum value during the end-inspiratory pause and then increases back to baseline during exhalation, which is also linked to the withdrawal of pressure support. Model simulations of right ventricular $SVI$ are directionally in agreement with the experimental data, although the simulated beat-to-beat changes are more pronounced in magnitude. Such a discrepancy cannot be sufficiently explained due to lack of RV $EDVI$ and $ESVI$ data as reported by [26]. However, in line with previous studies [29], the model-predicted intra-breath variation in RV $SVI$ (about 10 ml/m$^{2}$) is larger than the variation in LV $SVI$ (about 5 ml/m$^{2}$). This phenomenon is ascribed to the damping effect of the pulmonary circulation (i.e., pulmonary hydraulic impedance); namely, at every breath, for any given respiratory-induced increase in RV stroke volume, the corresponding increase in LV stroke volume is of smaller amplitude [29]. This is because the pulmonary circulation effectively accommodates for part of the blood volume that is ejected from the right ventricle before it reaches the left heart [30].

### C. Sensitivity Analysis

In the previous sections, we demonstrated the capability of the CP Model to replicate the physiological effects of mechanical ventilation on cardiac function. As a next step, we conduct a sensitivity analysis to examine the relative impact of the three highlighted model components (septum, pericardium, and pulmonary circulation model) on the CP Model’s capability to capture the heart-lung interactions during a breathing cycle. Such a sensitivity analysis complements the parameter sensitivity analysis conducted in [31].

For each of the three model components, we choose a key model parameter (physical property) for which a deviation from its nominal value would be indicative of a specific clinical pathology. For the pericardium, we choose the pericardial elastance ($k_{E, pcd}$ in Table I) because an increase in $k_{E, pcd}$ is associated with a stiffer pericardium, and thus indicative of constrictive pericarditis. For the septum, we choose the septal elastance ($k_{E, spt}$ in Table I) because an increase in $k_{E, spt}$ may be indicative of ventricular hypertrophy. Lastly, for the pulmonary circulation, we choose the total peripheral resistance of the pulmonary circulation ($R_{pp, tot}$ in (3)) since an increase in $R_{pp, tot}$ may represent a condition of pulmonary embolism.

We then simulate changes in the values of the aforementioned parameters ($k_{E, spt}$, $k_{E, pcd}$, or $R_{pp, tot}$) by setting them to 0.5 x baseline and 2 x baseline while examining stroke volume variations ($SVV$) of both left and right ventricles. $SVV$ is an index that is widely used clinically [28] to characterize the heart-lung interactions by summarizing the extent to which cardiac function is affected by mechanical ventilation. It is computed as:

\begin{equation*}
SVV = \frac{SV_{max} - SV_{min}}{(SV_{max} + SV_{min}) / 2} \cdot 100, \tag{5}
\end{equation*}

where $SV_{max}$ and $SV_{min}$ are the maximum and minimum $SV$ values within a breathing cycle, respectively.

Table IV and Table V present the values of LV and RV $SVV$ as the septal and pericardial elastances and the total pulmonary peripheral resistance are perturbed from their baseline values reported in Table I and Table II. We examined changes in the parameter values by 0.5 x baseline and 2 x baseline. Table IV and Table V also include the ratio (P/B, perturbed over baseline) between perturbed and baseline $SVV$ values. A P/B ratio larger (or smaller) than one indicates that the associated parameter change resulted in an increase (or decrease) in $SVV$.

**TABLE IV:** Effects of Septal and Pericardial Elastances on Ventricular Stroke Volume Variation

		LV $SVV$		RV $SVV$	
$k_{E, spt}$	$k_{E, pcd}$	Value (%)	P/B	Value (%)	P/B
0.5 x baseline	0.5 x baseline	13.92	1.20	21.85	1.10
	baseline	14.27	1.23	21.94	1.10
	2 x baseline	15.16	1.31	22.62	1.14
**baseline**	0.5 x baseline	11.30	0.97	19.42	0.98
	**baseline**	**11.59**	**—**	**19.86**	**—**
	2 x baseline	12.47	1.08	20.29	1.02
2 x baseline	0.5 x baseline	10.19	0.88	18.65	0.94
	baseline	10.31	0.89	18.75	0.94
	2 x baseline	11.06	0.95	19.18	0.97

Left and right ventricular stroke volume variation ($SVV$) are computed over a breathing cycle as septal ($k_{E, spt}$) and pericardial ($k_{E, pcd}$) elastances are perturbed from their baseline values, and ratio (P/B, perturbed over baseline) of perturbed LV and RV $SVV$ with respect to baseline.

**TABLE V:** Effect of Total Pulmonary Peripheral Resistance on Stroke Volume Variation

	LV $SVV$		RV $SVV$	
$R_{pp, tot}$	Value (%)	P/B	Value (%)	P/B
0.5 x baseline	12.40	1.07	19.65	0.99
**baseline**	**11.59**	**—**	**19.86**	**—**
2 x baseline	10.81	0.93	21.29	1.07

Left and right ventricular stroke volume variation ($SVV$) are computed over a breathing cycle as total pulmonary peripheral resistance ($R_{pp, tot}$) is perturbed from its baseline value, and ratio (P/B) of perturbed LV and RV $SVV$ with respect to baseline.

Table IV reinforces the fact that the septum has a predominant role in affecting left ventricular performance, which has also been supported by studies in the literature [11]. A twofold decrease in septal elastance ($k_{E, spt} = 0.5$ x baseline) causes an appreciable increase in LV $SVV$, while the same fold decrease in pericardial elastance ($k_{E, pcd} = 0.5$ x baseline) causes a decrease in LV $SVV$ (P/B of 1.23 vs 0.97, respectively). On the other hand, stiffening the pericardium ($k_{E, pcd}$ = 2 x baseline) compresses the pericardial space, hence resulting in larger intra-breath swings in stroke volume and a larger $SVV$. Table IV also shows that RV $SVV$ is less affected by changes in septal and pericardial elastances because it is primarily driven by the cyclic respiratory-induced variations in venous return (this physiological fact is also evident in the model). Table V demonstrates that changes in pulmonary peripheral resistance have minimal impact on LV and RV $SVV$. Nevertheless, it is valuable to note that changes in $R_{pp, tot}$ affect the two ventricles in opposite ways; that is, an increase in $R_{pp, tot}$ reduces LV $SVV$, but increases RV $SVV$. This is so since the pulmonary circulation is anatomically positioned between the right and left ventricles.

### D. Explanation of physiological phenomena

The following sections show how the CP Model can be used to explain physiologic phenomena that occur during mechanical ventilation conditions. First, we analyze the effects of PEEP on left and right ventricular functions via the Frank-Starling curves. Second, we illustrate the significance of the mechanical properties of the respiratory system in altering the effects of PEEP application on RV function and on the septum. This analysis also allows for the explanations of contradictory experimental results reported in the literature. Third, we demonstrate the intra-breath hemodynamic effects of mechanical ventilation on left and right ventricular preload, afterload, ejection fraction, and arterial pulse pressure.

#### 1) Frank-Starling Curves

**Figure 7.**
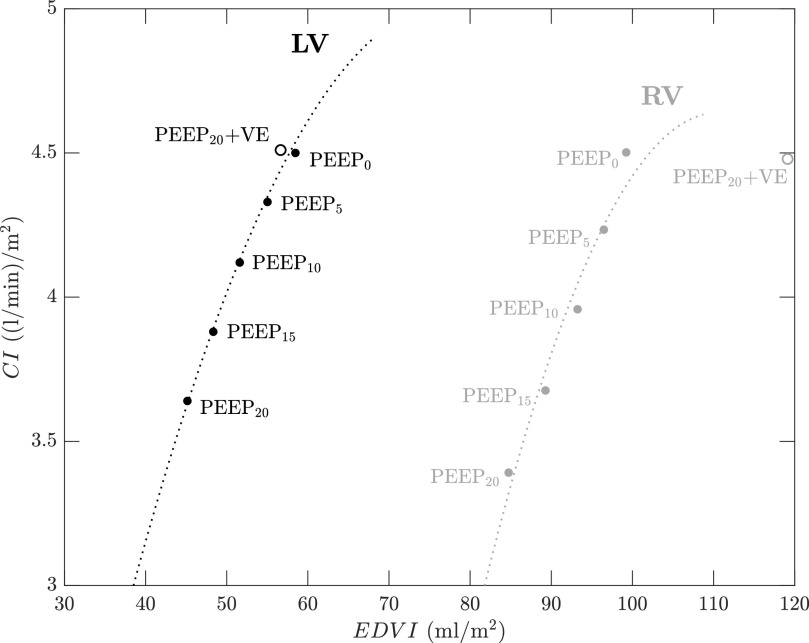
Model-simulated Frank-Starling curves describing cardiac index ($CI$) with end-diastolic volume index ($EDVI$) for left (*black* curve) and right (*gray* curve) ventricles. Filled circles indicate $CI$ versus $EDVI$ at different PEEP levels from 0 cmH$_{2}$O to 20 cmH$_{2}$O. Hollow circles represent $CI$ versus $EDVI$ at PEEP$_{20}$ and volume expansion (VE).

As shown in Figs. 4 and 5, the CP Model is able to capture the effect of PEEP on cardiac activity. The marked drop in the simulated cardiac output as PEEP is increased can be explained by the Frank-Starling mechanism and it is primarily due to a reduction in ventricular filling (preload). The decrease in preload is attributed to a reduction in venous return that is driven by the step increase in the external positive pressure. The underlying effects of the Frank-Starling mechanism on both left and right ventricular functions can be illustrated in Fig. 7. Fig. 7 presents left and right ventricular cardiac indices as functions of the corresponding $EDVI$ (preload) for all PEEP levels and it shows model outputs reproducing the Frank-Starling law. Note that, as mentioned in the Results & Validation section, volume expansion (VE) entirely reversed the output of both ventricles; namely, right and left cardiac indices at PEEP$_{20}$ + VE returned to about the same levels as those at PEEP$_{0}$. However, right ventricular $EDVI$ at PEEP$_{20}$ + VE was higher than its basal value (PEEP$_{0}$), an indication of right ventricular overload. This phenomenon resulted in the deviation of the “PEEP$_{20}$ + VE” point from the right ventricular function curve in Fig. 7.

Fig. 7 also reveals that the inotropic states of the two ventricles remain relatively unchanged with PEEP application since all respective points fall close to a fitted quadratic ventricular function curve (an increase in ventricular inotropy is indicated by a shift of the Frank-Starling curve upward and to the left). Further evidence of a constant inotropic states is provided by the model-predicted maximal ventricular elastances ($E_{max, lvf}$ and $E_{max, rvf}$). Both neural-modulated elastances attain values close to their basal conditions (at PEEP$_{0}$) for all PEEP levels; that is, $E_{max, lvf} = 4.544$, 4.540, 4.542, 4.554, and 4.577 mmHg/ml and $E_{max, rvf} = 0.723$, 0.721, 0.722, 0.729, and 0.742 mmHg/ml for PEEP$_{0}$, PEEP$_{5}$, PEEP$_{10}$, PEEP$_{15}$, and PEEP$_{20}$, respectively. These model predictions are in agreement with Huemer *et al.* [32] and Jardin *et al.* [33] who demonstrated that ventricular inotropy is independent of preload and constant over a wide range of afterload. In particular, they showed that changes in PEEP have moderate effects on the end-systolic left and right ventricular contractilities (i.e., inotropic states), despite the presence of some compensatory sympathetic activation due to the decrease in cardiac output (especially at high PEEP levels).

#### 2) PEEP Effects on RV Function and Septum

The mechanism of reduction in venous return due to an increase in PEEP is well established in the literature. However, contradictory results have been reported regarding the effects of PEEP on right ventricular volume. For instance, Dhainaut *et al.* [25] showed that a reduction in systemic venous return driven by a PEEP increase ultimately *reduces* right ventricular volumes (see RV $EDVI$ and $ESVI$ in Fig. 5). On the other hand, an earlier study by the same authors [27] as well as experimental data by Jardin *et al.* [30] had demonstrated an *increase* in the size of the right ventricle when PEEP was instituted. This phenomenon was attributed to an increase in pulmonary system impedance (RV afterload) that could, in turn, lead to right ventricular overloading (if excessive PEEP levels are applied) [27], [30].

A way to explain such contradictory experimental results is to investigate how changes in right ventricular function relate to the partitioning of the respiratory system elastance ($E_{rs}$) into lung ($E_L$) and chest wall components ($E_{cw}$). We hypothesize that the $E_{rs}$ partitioning alters the effects of PEEP on right ventricular preload and afterload. It is a known fact that for a given value of $E_{rs}$ ($E_{rs} = E_{L} + E_{cw}$), the same change in PEEP would result in different changes in pleural pressure ($P_{pl}$) depending on the ratio between $E_L$ and $E_{cw}$. Specifically, a low $E_L$ (high lung compliance) coupled with a high $E_{cw}$ would induce a notable increase in $P_{pl}$ as a response to an increase in PEEP level. In contrast, a high $E_L$ coupled with a low $E_{cw}$ would produce a smaller increase in $P_{pl}$ in response to the same PEEP variation. In addition, as previously illustrated, 1) right ventricular *preload* ($EDVI$) is influenced by pleural pressure via changes in systemic venous return, and 2) right ventricular *afterload* ($ESVI$) depends on the level of pulmonary capillary compression (pulmonary impedance) from lung expansion. As such, the different $E_{rs}$ partitioning could be the explanation behind the contradictory experimental results found in the studies by Dhainaut *et al.* [25] and Jardin *et al.* [27], [30].

**Figure 8.**
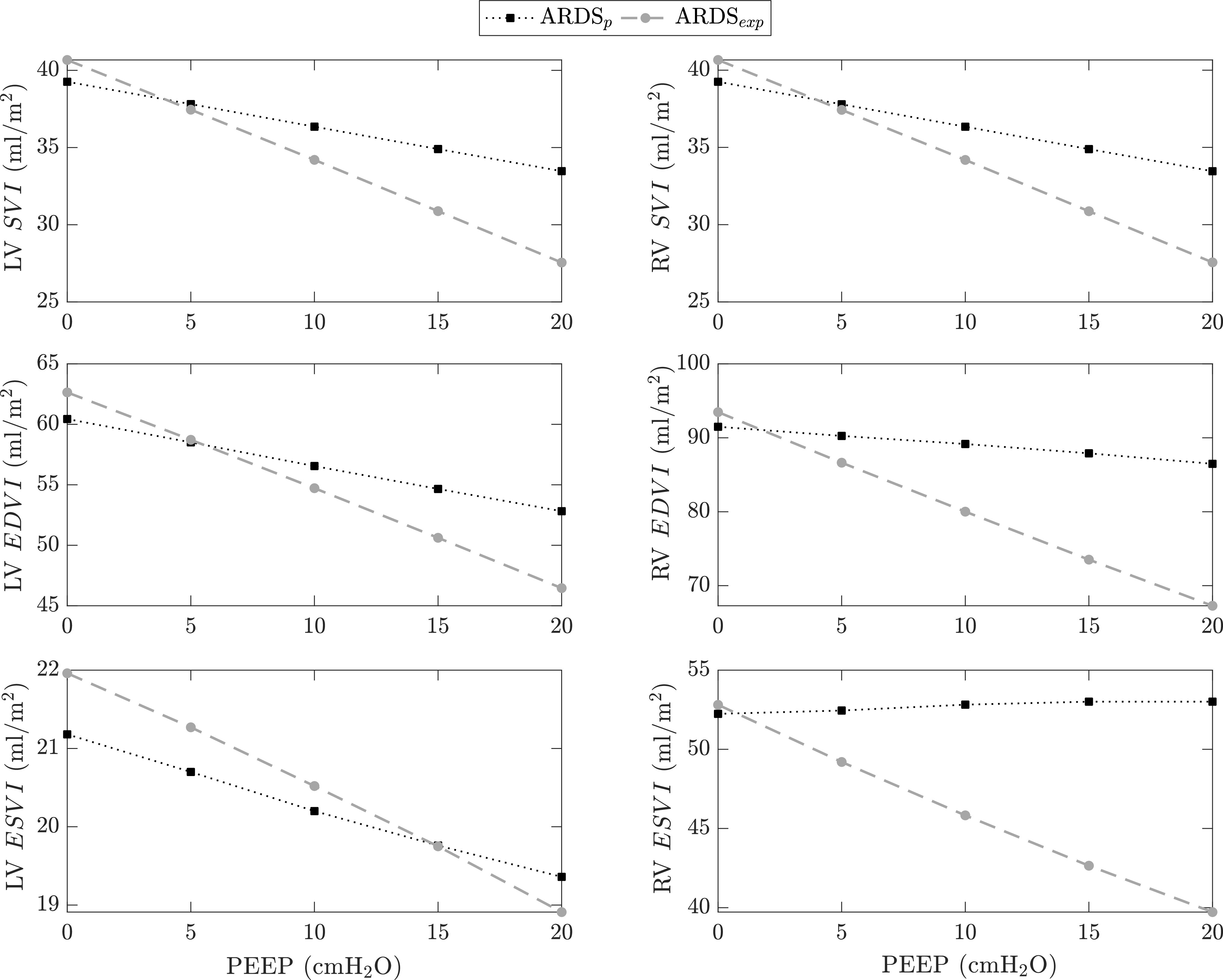
Cardiovascular response to step changes in the level of positive end-expiratory pressure (PEEP) of a virtual patient with either pulmonary ARDS (ARDS$_{p}$, *black* squares) or extra-pulmonary ARDS (ARDS$_{exp}$, *gray* circles). Each PEEP level is applied for a period of 20 minutes and simulation results are averaged over the last 10 minutes of each PEEP segment. LV, left ventricular; RV, right ventricular; $SVI$, stroke volume index; $EDVI$, end-diastolic volume index; $ESVI$, end-systolic volume index.

To investigate this hypothesis, we set up our CP Model to simulate the response of subjects with different $E_{rs}$ partitioning into $E_L$ and $E_{cw}$. In the literature, profound differences in the $E_{rs}$ partitioning have been reported between two groups of ARDS patients: 1) ARDS patients with pulmonary diseases such as pneumonia (ARDS$_{p}$), and 2) ARDS patients with extra-pulmonary diseases such as peritonitis (ARDS$_{exp}$). In a study by Gattinoni *et al.* [34], the ARDS$_{p}$ group had $E_L = 20.23$ cmH$_{2}$O/l and $E_{cw} = 5.31$ cmH$_{2}$O/l, whereas the ARDS$_{exp}$ group had $E_L$ = 15.95 cmH$_{2}$O/l and $E_{cw}$ = 15.88 cmH$_{2}$O/l (all values are at zero PEEP), with $E_{rs}$ being approximately the same between the two groups. Based on this, we use the CP Model to simulate the response of the two ARDS groups (ARDS$_{p}$ and ARDS$_{exp}$) under a PEEP-step protocol. Fig. 8 compares the simulated cardiovascular responses (LV and RV $SVI$, $EDVI$, and $ESVI$) for ARDS$_{p}$ (black squares) and ARDS$_{exp}$ (gray circles) as PEEP is increased from 0 to 20 cmH$_{2}$O in steps of 5 cmH$_{2}$O. The notable discrepancy in the direction of change of the RV $ESVI$ as PEEP increases (lower right subfigure of Fig. 8) clearly illustrates that the different $E_{rs}$ partitioning between ARDS$_{p}$ and ARDS$_{exp}$ alters the effect of PEEP on the loading status of the right ventricle. It is clear from simulation that right ventricular $ESVI$ decreases for ARDS$_{exp}$ due to a marked drop in $EDVI$, whereas it increases for ARDS$_{p}$. The increase in RV $ESVI$ for ARDS$_{p}$ is a direct consequence of 1) the increased right ventricular afterload (pulmonary impedance) that is caused by the compression of the pulmonary peripheral vessels, and 2) the moderate decrease in RV preload ($EDVI$). The small decrease in $EDVI$ is attributed to the combination of stiff lungs (high $E_L$) with a more compliant chest wall (low $E_{cw}$) that characterizes the ARDS$_{p}$ condition. It is worth noting that these opposite RV $ESVI$ responses between ARDS$_{p}$ and ARDS$_{exp}$ occur despite the overall decrease in systemic venous return that causes a reduction in left and right ventricular $EDVI$ and $SVI$ in both cases. These observations then suggest that institution of PEEP on a patient with ARDS$_{p}$ may lead to right ventricular overloading, potentially causing right ventricular failure [35], despite the overall decrease in systemic venous return.

Contradictory experimental findings have also been reported regarding the movement of the septum in response to changes in PEEP. Some researchers, like Jardin *et al.* [30], [33], demonstrated that PEEP application increases the septal curvature by shifting the interventricular septum leftwards (the curvature is an indication of the position of the septum inside the heart). Such a septal movement effectively constricts the left ventricle, thereby reducing left ventricular filling and ejection capacity. In contrast, studies by Dhainaut *et al.* [27] and Huemer *et al.* [32] showed negligible ventricular interdependence with a minimal change in the radius of the septal curvature. To explain such contradictory observations, again, we simulated results for ARDS$_{p}$ and ARDS$_{exp}$. In the ARDS$_{p}$ case, the model outputs show that the increased right ventricular afterload ($ESVI$) after a PEEP increase reduces the septal volume, effectively pushing the septum toward the left ventricular free wall. Specifically, $\bar{V}_{spt} = 2.16$, 2.12, 2.06, 1.99, and 1.93 ml for PEEP = 0, 5, 10, 15, and 20 cmH$_{2}$O, respectively, where $\bar{V}_{spt}$ is the average septal volume over the last 10 minutes of each PEEP interval. In contrast, the ARDS$_{exp}$ simulations show an increase in septal volume from 2 ml at PEEP$_{0}$ to 2.37 ml at PEEP$_{20}$, indicating a septal movement toward the right ventricular free wall. This direction in the movement of the septum is attributed to the marked reduction in the right ventricular $EDVI$ in the ARDS$_{exp}$ case (see Fig. 8).

#### 3) Intra-Breath Hemodynamic Effects of Mechanical Ventilation

**Figure 9.**
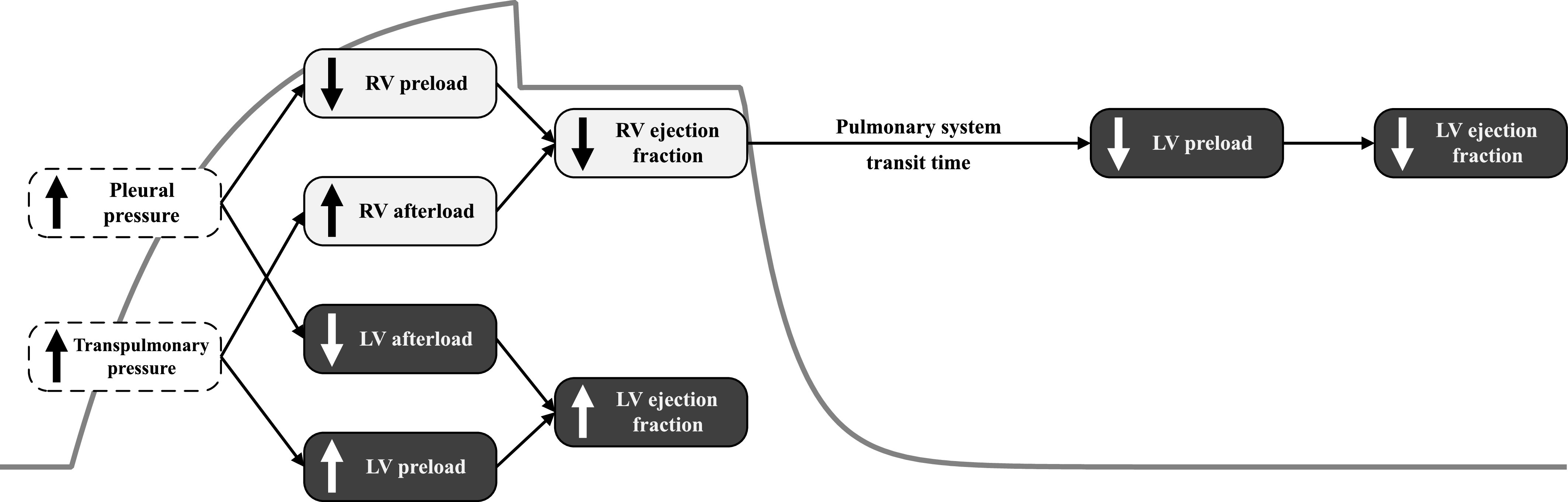
Hemodynamic effects of mechanical ventilation in relation to changes in airway opening pressure (gray waveform). *White* dashed boxes indicate changes in respiratory system variables, whereas *light* and *dark gray* boxes respectively refer to changes in right (RV) and left (LV) ventricular functions. Transpulmonary pressure is defined as the difference between alveolar and pleural pressures. The figure is adapted from [28].

**Figure 10.**
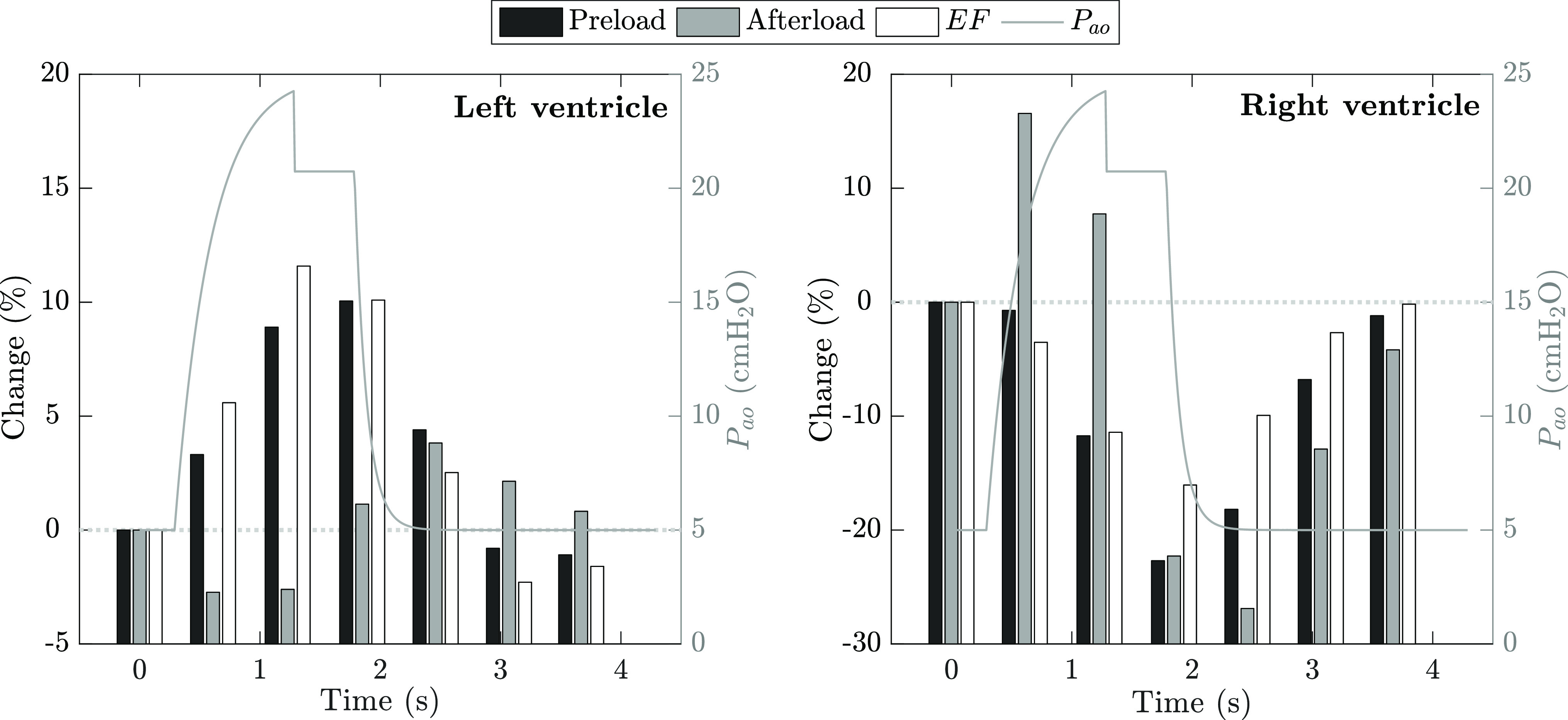
Percent change (left vertical axes) in left (*left* plot) and right (*right* plot) ventricular preload (*black* bars), afterload (*gray* bars), and ejection fraction (*white* bars) during a breathing cycle (P$_{ao}$, right vertical axes). Ventricular function indices have been computed based on the simulation results in Fig. 6 as follows: preload $:= EDVI$; afterload $:=$ ventricular wall stress, $\sigma \propto P \cdot \sqrt[3]{V}$, where $P$ is the end-systolic transmural pressure and $V$ is the corresponding end-systolic volume [32]; ejection fraction, $EF = SVI/EDVI$. The gray horizontal dotted line indicates the zero percent level of the change in preload, afterload, or ejection fraction.

In the previous sections, we described heart-lung interaction phenomena during PEEP application. We now focus on the hemodynamic effects of mechanical ventilation over a breathing cycle. Ventricular function is usually described by three indicators: preload, afterload, and ejection fraction. Preload is defined as the level of stretching of the cardiac myocytes immediately before contraction. Afterload is the maximal stress applied on the ventricular wall during contraction and is associated with the load that the ventricle needs to overcome to eject blood. Finally, ejection fraction is the proportion of blood pumped by a ventricle per cardiac cycle, and depends on both preload and afterload. Positive pressure ventilation is known to affect all three indicators. For instance, Fig. 9 qualitatively summarizes, based on experimental evidence by Michard and Teboul [28], the changes induced by mechanical ventilation on left and right ventricular functions. Fig. 10 depicts beat-to-beat changes in preload, afterload, and ejection fraction as simulated by our CP Model. It is evident that the simulations in Fig. 10 are qualitatively in good agreement with Fig. 9. We know that positive pressure inhalation induces lung expansion by increasing transpulmonary and pleural pressures. We then observe that these positive swings in pleural pressure reduce right ventricular preload via a reduction in systemic venous return (see Fig. 9 and black bars in right plot of Fig. 10). Such pleural pressure swings also increase pericardial pressure, which prompts a decrease in left ventricular transmural pressure. Since afterload depends on the pressure across the ventricular wall, a reduction in transmural systolic pressure lowers the afterload of the left ventricle (see Fig. 9 and gray bars in left plot of Fig. 10). At the same time, inspiratory elevation of alveolar pressure and compression of the pulmonary peripheral vessels due to lung inflation have two consequences: 1) an increase in RV afterload (due to an increase in pulmonary system impedance, see Fig. 9 and gray bars in right plot of Fig. 10), and 2) an increase in LV preload (due to the compression of the pulmonary peripheral vessels which promotes blood flow into the left ventricle, see Fig. 9 and black bars in left plot of Fig. 10). As for the ejection fraction, the decrease in right ventricular preload during inhalation, along with the concomitant increase in right ventricular afterload, generates a reduction in right ventricular ejection fraction (see Fig. 9 and white bars in right plot of Fig. 10). In addition, the increased preload of the left ventricle (accompanied by a decrease of its afterload) generates a transient increase in LV ejection fraction toward the end of inhalation (see Fig. 9 and white bars in left plot of Fig. 10). During exhalation, the inspiratory reduction in right ventricular ejection lowers the filling of the left ventricle which, in turn, reduces LV stroke volume and ejection fraction as per the Frank-Starling mechanism.

#### 4) Intra-Breath Variations in Pulse Pressure

Intra-breath variations in cardiac activity, such as those summarized in Figs. 9 and 10, result in changes in stroke volume, and consequently in arterial pulse pressure (pulse pressure ($PP$) is considered to be proportional to stroke volume assuming a constant arterial compliance). Since $PP$ is monitored at the bedside, intra-breath variations in $PP$ have been proposed as dynamic predictors to two clinical interventions: PEEP application [36] and fluid resuscitation (volume expansion) [37], [38]. Both interventions, though distinct, share the same underlying mechanism affecting cardiac activity; they induce a change in preload (negative for PEEP application, positive for volume expansion) that leads to a shift in the heart’s operating point on the Frank-Starling curve (see Fig. 7). Such a shift effectively alters the variation in pulse pressure for any given intra-breath change in cardiac preload ($EDVI$).

Given the clinical relevance of PEEP and volume expansion (VE) therapies, we simulate them both using the CP Model. We measure the percent change in pulse pressure over a breathing cycle as originally proposed by Michard *et al.* [36], [38]:

\begin{equation*}
\Delta PP = \frac{PP_{max} - PP_{min}}{(PP_{max} + PP_{min}) / 2} \cdot 100, \tag{6}
\end{equation*}

where $PP_{max}$ and $PP_{min}$ are the maximum and minimum pulse pressure values within a breathing cycle, respectively. Following our analysis on stroke volume (see Fig. 10), we expect that $PP_{max}$ occurs at peak inspiration whereas $PP_{min}$ during exhalation. For both PEEP and volume expansion scenarios, we simulate two patient groups with different pathologies: one with low blood volume (hypovolemia) and one with low systemic vascular resistance (e.g., sepsis). Considering that both pathologies are clinically manifested by low blood pressure (hypotension), we select the model parameters such that the two groups have the same (low) baseline mean arterial blood pressure. The model predictions in terms of $\Delta PP$ demonstrate a good agreement with experimental findings from literature studies [36], [38]; namely, a high $\Delta PP$ value, prior to intervention (whether PEEP or VE therapy), is indicative of a hypovolemic subject.

Table VI summarizes the results of the simulation studies. The *septic* virtual patient in the first scenario (PEEP application) has a $\Delta PP$ of about 9.5% with an average cardiac index of 4.15 (l/min)/m$^{2}$ at zero PEEP. On the other hand, in the *hypovolemic* case, $\Delta PP$ is initially 15.5% and $CI$ is 3.15 (l/min)/m$^{2}$. As expected, the hypovolemic patient has a lower cardiac index due to a reduced overall blood volume, and thus reduced cardiac preload. When PEEP of 10 cmH$_{2}$O is applied, the *hypovolemic* subject shows a decrease in $CI$ of about 10% to 2.83 (l/min)/m$^{2}$ ($\Delta PP$ increases to 19.2%), while the cardiac index of the *septic* virtual patient reduces by only 2.6% to 4.04 (l/min)/m$^{2}$ ($\Delta PP$ increases to just 9.9%). Obviously, there is a correlation between $\Delta PP$ at zero PEEP and the magnitude of change in cardiac index after a PEEP increase. This is in agreement with what was observed in [36]; namely, the higher the $\Delta PP$ at zero PEEP, the larger the drop in $CI$. It is also worth noting that the simulated increase in $\Delta PP$ in both hypovolemic and septic cases after PEEP application is in agreement with the experimental results reported by Michard *et al.* [36] and Kubitz *et al.* [39]. Such an increase is due to the fact that PEEP reduces cardiac volumes, effectively forcing the heart to work on a steeper portion of the Frank-Starling curve (see Fig. 7, for example). Thus, the magnitude of change in stroke volume and in pulse pressure ($\Delta PP$) for any given change in left ventricular filling (preload) depends on the level of PEEP; the higher the PEEP, the larger the $\Delta PP$, as also indicated by [40].

**TABLE VI:** $\Delta PP$ as Clinical Predictor of PEEP Application and Volume Expansion (VE) Therapies

	$\Delta PP$ $(\%)$		$CI$ ((l/min)/m$^{2}$)		$\Delta CI$ $(\%)$
	PEEP$_{0}$	PEEP$_{10}$	PEEP$_{0}$	PEEP$_{10}$	
Hypovolemic	15.5	19.2	3.5	2.83	$-10$
Septic (normovolemic)	9.5	9.9	4.15	4.04	$-2.6$
	Before VE	After VE	Before VE	After VE	
Hypovolemic	18.2	11.7	2.93	3.41	16.4
Septic (normovolemic)	9.8	7.5	4.1	4.48	9.2

$\Delta PP$, percent change in pulse pressure over a breathing cycle; $CI$, cardiac index; $\Delta CI$, percent change in $CI$ before and after the intervention; PEEP $_{x}$, PEEP at x cmH$_{2}$O where x = {0, 10}.

Similar conclusions on $\Delta PP$ can be drawn for the fluid resuscitation (or volume expansion, VE) scenario; that is, the higher the $\Delta PP$ before VE, the larger the effect of fluid resuscitation in augmenting $CI$ (see Table VI). For the *hypovolemic* case, $\Delta PP$ and $CI$ are initially (i.e., before VE) 18.2% and 2.93 (l/min)/m$^{2}$, respectively. After a 500 ml fluid administration (similar to the experimental protocol in [38]), $\Delta PP$ reduces to 11.7%, while $CI$ increases to 3.41 (l/min)/m$^{2}$, a 16.4% increase. In contrast, the *septic* virtual patient shows much smaller changes in both $\Delta PP$ and $CI$; $\Delta PP$ is initially 9.8% and then reduces to 7.5%, while cardiac index increases by only 9.2%, from 4.1 to 4.48 (l/min)/m$^{2}$. We can therefore conclude that the model predictions adhere to the 13% threshold in the $\Delta PP$ value before VE, which allowed Michard *et al.* [38] to discriminate between responders (increase in $CI$ after VE greater than 15%) and non-responders with 94% sensitivity and 96% specificity.

## IV. Discussion

In this study, we have developed an integrated cardiopulmonary model to 1) analyze heart-lung interactions during mechanical ventilation, and 2) evaluate effects of those interactions on cardiac activity. Mechanical ventilation is typically instituted as a life-saving therapy, however, it can profoundly compromise cardiac performance. Mathematical models of the cardiopulmonary physiology can be especially useful to interpret the interactions between heart and lungs and to analyze the potential negative effects of mechanical ventilation therapy on cardiac function. The cardiopulmonary model (CP Model) presented in this paper captures the main mechanisms of cardiorespiratory interactions and it includes a pericardial membrane, an interventricular septum, and a pulmonary circulation model that accounts for the effects of pulmonary capillary compression during inhalation (see Figs. 2 and 3). The CP Model was validated with patient data in normal resting conditions (see Table III) and under mechanical ventilation scenarios (see Figs. 4–6). Ventilation conditions were simulated via a simple ventilator model able to replicate common ventilator settings, such as pressure control level, PEEP, and inspiration-to-expiration ratio. The ventilator model includes all necessary elements to simulate any type of ventilation modes, such as pressure-control ventilation (PCV), pressure support ventilation (PSV), and volume-control ventilation (VCV). Despite the proven capability of the proposed model in describing heart-lung interaction mechanisms, it is necessary to point out some limitations. These can also serve as a basis for future work.

First, all scenarios and validation processes presented in this paper pertain to passively breathing subjects. Indeed, spontaneously breathing subjects under mechanical ventilation support exhibit more elaborate dynamics due to neural and mechanical reflexes. Nevertheless, the present CP Model due to its basis on the work by Albanese *et al.* [7], [31] includes a comprehensive neural control module. The neural component models short-term neural control mechanisms acting on both the cardiovascular and the respiratory functions, such as baroreceptors, peripheral and central chemoreceptors as well as lung-stretch receptors. An example of the capability of the model to simulate spontaneously breathing subjects has been presented in [12].

**Figure 11.**
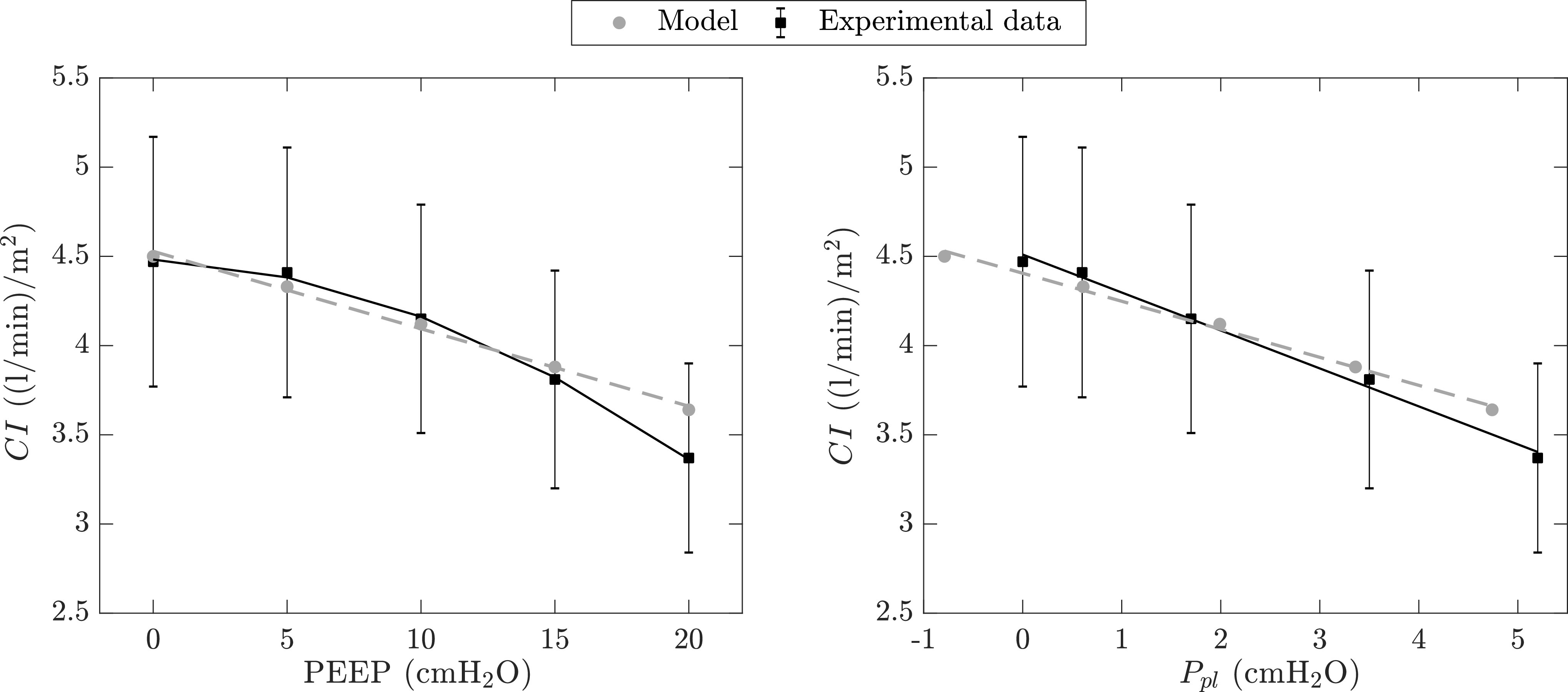
Comparison of the effects of positive end-expiratory pressure (PEEP, *left* plot) and pleural pressure ($P_{pl}$, *right* plot) on cardiac index ($CI$) between model-predicted (*gray* circles) and experimental (*black* squares) data. Experimental data are taken from Dhainaut *et al.* [25] and are reported with their mean and standard error of the means. Notice that each $P_{pl}$ point represents the average pleural pressure (or its surrogate, esophageal pressure) at each corresponding PEEP level.

Second, the CP Model does not consider the effects of PEEP on alveolar recruitment and gas exchange. For instance, it is well known that institution of PEEP on ARDS patients is recommended to improve gas exchange by inflating the collapsed alveoli and reducing edema and intrapulmonary shunt [41]. Alveolar recruitment is typically modeled via a nonlinear pressure-volume relationship [42], where lung compliance increases as the collapsed lung regions are being recruited with PEEP application. Consequently, the recruited alveoli can participate in gas exchange, effectively prompting a reduction in intrapulmonary shunt. However, the proposed model assumes a linear pressure-volume relationship (hence, constant lung compliance) and a constant value for the shunt fraction. Besides gas exchange, the assumption of a constant lung compliance has disadvantages regarding the mechanism that describes the effects of PEEP on cardiac output (see Fig. 11). Specifically, Dhainaut *et al.* [25] found a curvilinear relationship between PEEP and cardiac index (black squares in left plot of Fig. 11), whereas our model predicts a linear relationship between the two (gray circles in left plot of Fig. 11). At the same time, $CI$ and pleural pressure are linearly related for both simulated and experimental data (right plot in Fig. 11). It is worth noticing, however, the effect of PEEP on pleural pressure. Each step increase in PEEP results in a constant step increment in pleural pressure in our CP Model, whereas the experimental data show larger step increments at higher PEEP levels (compare the x-axis increments in the right hand-side plot of Fig. 11). Like Dhainaut *et al.* [25], we conjecture that the varying step increments in pleural pressure with each PEEP increase are attributed to a nonlinear lung compliance. The increased compliance at higher PEEP levels allows a larger lung expansion. This, in turn, leads to a larger compression of the pleural space which translates into a larger increase in $P_{pl}$ compared to that at low PEEP. Based on these observations, we can conclude that since pleural pressure directly affects venous return, the nonlinear lung compliance and the varying step increments in $P_{pl}$ after each PEEP increase are responsible for the curvilinear relationship between $CI$ and PEEP that is reported in literature [25].

Third, some limitations also exist in the heart model. 1) As indicated in the Methods section, the proposed CP Model does not consider any mechanical interdependence between the two atria [43]. 2) Furthermore, a constant left ventricular diastolic pressure-volume relationship is used. Although studies [30], [44] have shown that right ventricular overload may lead to a reduction in left ventricular diastolic elastance, the current model implementation only considers the leftward movement of the septum as the sole reason for right-to-left ventricular interference that causes a decrease in left ventricular volume. 3) Finally, activations of the left and right ventricular free walls are assumed to happen simultaneously. This assumption is based on the absence of concrete experimental evidence regarding the delay between left and right ventricular contractions in healthy individuals [45]–[47]. Nevertheless, we have included a provision in our model to simulate contraction delays due to pathological conditions, such as left or right bundle branch blocks.

Lastly, it is known that systemic venous return depends on the pressure gradient between the extra-thoracic veins (upstream pressure) and the venae cavae inside the thorax (downstream pressure). These two pressure points are, in turn, affected by abdominal and pleural pressures, respectively. While mechanical ventilation induces positive swings in pleural pressure, it also causes the diaphragm to descend, thereby raising abdominal pressure [48], [49]. Consequently, while pleural pressure swings are considered the primary determinants in decreasing venous return, the concomitant increase in abdominal pressure is expected to minimize the effect of $P_{pl}$ in reducing venous return. In our model, we assume abdominal pressure equal to atmospheric pressure (zero). Thus, any simulated changes in venous return depend solely on changes in pleural pressure, potentially resulting in model simulations with larger than expected decrease in venous return as compared with experimental data.

## V. Conclusion

In conclusion, we have hereby presented a mathematical cardiopulmonary model with a pericardial membrane, an interventricular septum, and a pulmonary circulation model that accounts for the compression of the pulmonary peripheral vessels due to lung inflation. Such a model allows for a better understanding of heart-lung interactions during mechanical ventilation. For instance, the inclusion of the pericardium allows to simulate cardiac diseases, such as pericarditis and cardiac tamponade. The model was validated with experimental data, both in transient (intra-breath) and steady-state conditions (PEEP application). Moreover, model simulations were used to provide physiologic explanations to a few contradictory experimental observations, thus proving the potentials of such a model to improve the current understanding of complex physiological phenomena. We therefore believe that this CP Model can serve as a tool to study, analyze, and evaluate the effects of mechanical ventilation therapy on cardiac function, contributing in making such a therapy safer for patients, including COVID-19 patients who require intubation due to their comorbidities and who may suffer of a high risk of cardiovascular complications.

## Appendix Interventricular septum

The septal elastance effector response to the ANS sympathetic activity is modeled according to the approach proposed by Ursino and Magosso [17]. Such a model includes a logarithmic function to describe the effector’s static response, a pure delay, and a low-pass first-order filter to simulate its dynamic behavior. Specifically, the maximal elastance of the septum ($E_{max, spt}$) changes with respect to the frequency of the sympathetic efferent fibers ($f_{sh}$) according to the following equations:

\begin{align*}
&\sigma _{E_{max, spt}}\\
& = {\begin{cases}G_{E_{max, spt}} \cdot \ln \left(f_{sh} \cdot (t - D_{E_{max, spt}}) - f_{es, min} + 1 \right), \\
{\kern70.0pt} \text{if } f_{sh} \geq f_{es, min} \\
0, {\kern61.00006pt} \text{if } f_{sh} < f_{es, min} \end{cases}}, \tag{A1} \\
&\frac{d \Delta E_{max, spt}}{dt} = \frac{1}{\tau _{E_{max, spt}}} \cdot \left(- \Delta E_{max, spt} + \sigma _{E_{max, spt}}\right) \text{, and} \tag{A2} \\
&E_{max, spt}(t) = \Delta E_{max, spt}(t) + E_{max, spt0}, \tag{A3}
\end{align*}

where $\sigma _{E_{max, spt}}$ is the output of the logarithmic static function of (A1) that is used to calculate the maximal septal elastance in (A3), $G_{E_{max, spt}}$ is a gain factor, $D_{E_{max, spt}}$ is the latency in the static response, $\tau _{E_{max, spt}}$ is the time constant of the first-order filter, and $f_{es, min}$ is a threshold for sympathetic stimulation. Following the method by Ursino and Magosso [17], $G_{E_{max, spt}}$ and $E_{max, spt0}$ are respectively set to 16.1% and 81% of the original septal elastance value reported in [16]. The parameter values, besides $E_{max, spt0}$ (see Table I), of this reflex regulatory mechanism are reported in Table VII.

**TABLE VII:** Parameters of the Septal Elastance Reflex Effector Model

$D_{E_{max, spt}}$ = 2 s [17]	$\tau _{E_{max, spt}}$ = 8 s [17]
$G_{E_{max, spt}}$ = 6.44 mmHg$\cdot$ml$^{-1}$$\cdot$(spikes/s)$^{-1}$	$f_{es, min}$ = 2.66 spikes/s [17]

See text for explanation of symbols.

## Pulmonary peripheral vessels

The equations describing the pulmonary circulation model are obtained by applying the conservation of mass (continuity equation) and momentum (compatibility equation) laws on the electrical analog in Fig. 3.

\begin{align*}
C_{pa} \cdot \frac{d \left(P_{pa} - P_{pl}\right)}{dt} =& Q_{rv,o} - Q_{pa} \tag{A4}\\
V_{pa} =& C_{pa} \cdot \left(P_{pa} - P_{pl}\right) + V_{u,pa} \tag{A5}\\
L_{pa} \cdot \frac{d Q_{pa}}{dt} =& P_{pa} - P_{pal} - R_{sa} \cdot Q_{pa} \tag{A6}\\
C_{pal} \cdot \frac{d \left(P_{pal} - P_{pl}\right)}{dt} =& Q_{pa} - (Q_{pal} + Q_{ps}) \tag{A7}\\
Q_{pp, tot} =& Q_{pal} + Q_{ps} = \frac{Q_{pal}}{1 - sh}\tag{A8}\\
Q_{pal} =& \frac{P_{pal} - P_{pc}}{R_{pal}} \tag{A9}\\
V_{pal} =& C_{pal} \cdot \left(P_{pal} - P_{pl}\right) + V_{u, pal} \tag{A10}\\
C_{pc} \cdot \frac{d \left(P_{pc} - P_A\right)}{dt} =& Q_{pal} - Q_{pc} \tag{A11}\\
Q_{pc} =& \frac{P_{pc} - P_{pv}}{R_{pc}} \tag{A12}\\
V_{pc} =& C_{pc} \cdot \left(P_{pc} - P_A\right) + V_{u, pc} \tag{A13}\\
C_{pv} \cdot \frac{d \left(P_{pv} - P_{pl}\right)}{dt} =& Q_{ps} + Q_{pc} - Q_{pv} \tag{A14}\\
Q_{ps} =& \frac{sh}{1 - sh} \cdot Q_{pal} \tag{A15}\\
Q_{pv} =& \frac{P_{pv} - P_{la}}{R_{pv}} \tag{A16}\\
V_{pv} =& C_{pv} \cdot \left(P_{pv} - P_{pl}\right) + V_{u, pv} \tag{A17}
\end{align*}

Equation (3), which defines the $V_A$-dependent arteriolar resistance, is based on the equation proposed by Lu *et al.* [10]:

\begin{equation*}
R_{pal} (V_A) = R_{pal,0} \cdot \left(\frac{V_A}{V_{A, max}} \right)^2, \tag{A18}
\end{equation*}

where $R_{pal,0}$ is a constant value that represents the arteriolar resistance when alveolar volume has reached its maximum value $V_{A, max}$. However, (A18), as originally proposed by Lu *et al.* [10], does not account for the pulmonary shunt (the amount of blood that does not contribute to gas exchange) that is included in the pulmonary circulation model of our CP Model (see Figs. 2 and 3). To introduce the effects of anatomical shunting in our model, we consider that the resistances of shunted ($R_{ps}$) and non-shunted ($R_{pal}$ and $R_{pc}$) pulmonary peripheral compartments are functions of the selected shunt fraction ($sh$) such that the distribution of blood between the two segments matches the $sh$ value ($sh$ is equal to 1.7% in normal physiological conditions). Derivation of (3) from (A18) is based on the following two assumptions:

1) $R_{pp, tot}$ is the total resistance of the pulmonary peripheral circulation, i.e., the equivalent resistance of the electrical circuit between $P_{pal}$ and $P_{pv}$ in Fig. 3, at steady-state conditions and at a nominal functional residual capacity, $FRC_{nom}$,
  \begin{equation*}
  R_{pp, tot} = \frac{R_{ps} \cdot \left(R_{pc} + R_{pal@FRC_{nom}} \right)}{R_{ps} + R_{pc} + R_{pal@FRC_{nom}}}, \tag{A19}
  \end{equation*}
  where $R_{pal@FRC_{nom}}$ is the value of the $V_A$-dependent arteriolar resistance in (A18) at $V_A = FRC_{nom}$.
2) Similar to the model by Lu *et al.* [10], the resistance $R_{pc}$ does not change with respect to $V_A$. Its value is considered equal to $R_{pal}$ when alveolar volume is equal to $FRC_{nom}$,
  \begin{equation*}
  R_{pc} = R_{pal@FRC_{nom}}. \tag{A20}
  \end{equation*}

Consequently, at steady-state conditions when $V_A = FRC_{nom}$,

\begin{align*}
\frac{Q_{pal}}{Q_{ps}} =& \frac{1 - sh}{sh} \Rightarrow R_{ps} = \frac{1 - sh}{sh} \cdot \left(R_{pal@FRC_{nom}} + R_{pc} \right) \\
=& \frac{2 \cdot (1 - sh) \cdot R_{pal@FRC_{nom}}}{sh}. \tag{A21}
\end{align*}

Hence, (A19) becomes:

\begin{equation*}
R_{pp, tot} = 2 \cdot (1 - sh) \cdot R_{pal@FRC_{nom}}. \tag{A22}
\end{equation*}

Using the definition for $R_{pal}$ in (A18), (A22) gives us the constant, but unknown, quantity $R_{pal, 0}$ as:

\begin{equation*}
R_{pal, 0} = \frac{R_{pp, tot}}{2 \cdot (1 - sh)} \cdot \left(\frac{V_{A, max}}{FRC_{nom}} \right)^2. \tag{A23}
\end{equation*}

Then,

\begin{align*}
R_{pal} =& \frac{R_{pp, tot}}{2 \cdot (1 - sh)} \cdot \left(\frac{V_{A}}{FRC_{nom}} \right)^2, \tag{A24}\\
R_{pc} =& \frac{R_{pp, tot}}{2 \cdot (1 - sh)} . \tag{A25}
\end{align*}

Note that the shunt resistance $R_{ps}$ does not need to be explicitly specified because the flow over the pulmonary shunts, $Q_{ps}$, is replaced by $Q_{pal}$ according to (A15).
